# Well-defined silica supported aluminum hydride: another step towards the utopian single site dream?†
†Electronic supplementary information (ESI) available. See DOI: 10.1039/c5sc02276b



**DOI:** 10.1039/c5sc02276b

**Published:** 2015-07-20

**Authors:** Baraa Werghi, Anissa Bendjeriou-Sedjerari, Julien Sofack-Kreutzer, Abdesslem Jedidi, Edy Abou-Hamad, Luigi Cavallo, Jean-Marie Basset

**Affiliations:** a KAUST Catalysis Center (KCC , ) , King Abdullah University of Science and Technology , Thuwal-23955-6900 , Saudi Arabia . Email: luigi.cavallo@kaust.edu.sa ; Email: jeanmarie.basset@kaust.edu.sa

## Abstract

Reaction of triisobutylaluminum with SBA15_700_ at room temperature occurs by two parallel pathways involving either silanol or siloxane bridges. It leads to the formation of a well-defined bipodal [(≡SiO)_2_Al–CH_2_CH(CH_3_)_2_] **1a**, silicon isobutyl [≡Si–CH_2_CH(CH_3_)_2_] **1b** and a silicon hydride [≡Si–H] **1c**. Their structural identity was characterized by FT-IR and advanced solid-state NMR spectroscopies (^1^H, ^13^C, ^29^Si, ^27^Al and 2D multiple quantum), elemental and gas phase analysis, and DFT calculations. The reaction involves the formation of a highly reactive monopodal intermediate: [≡SiO–Al–[CH_2_CH(CH_3_)_2_]_2_], with evolution of isobutane. This intermediate undergoes two parallel routes: transfer of either one isobutyl fragment or of one hydride to an adjacent silicon atom. Both processes occur by opening of a strained siloxane bridge, ≡Si–O–Si≡ but with two different mechanisms, showing that the reality of “single site” catalyst may be an utopia: DFT calculations indicate that isobutyl transfer occurs *via* a simple metathesis between the Al-isobutyl and O–Si bonds, while hydride transfer occurs *via* a two steps mechanism, the first one is a β-H elimination to Al with elimination of isobutene, whereas the second is a metathesis step between the formed Al–H bond and a O–Si bond. Thermal treatment of **1a** (at 250 °C) under high vacuum (10^–5^ mbar) generates Al–H through a β-H elimination of isobutyl fragment. These supported well-defined Al–H which are highly stable with time, are tetra, penta and octa coordinated as demonstrated by IR and ^27^Al–^1^H J-HMQC NMR spectroscopy. All these observations indicate that surfaces atoms around the site of grafting play a considerable role in the reactivity of a single site system.

## Abstract 2

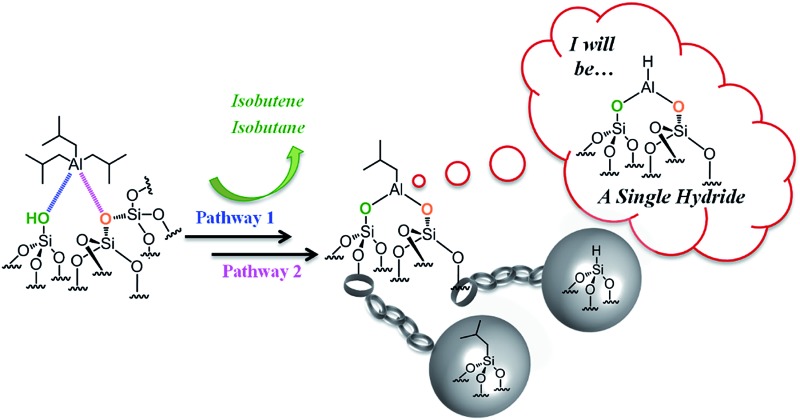
Reaction of triisobutylaluminum with SBA15_700_ at room temperature occurs by two parallel pathways involving either silanol or siloxane bridges.

## Introduction

There is a tendency to rationalize the understanding of heterogeneous catalysis by making well-defined model compounds.^1–8^ The study of the reactivity of these models allows the identification of some elementary steps of heterogeneous catalysis.^9–14^ One of the simplest ways to make such models compounds is to react the surface silanols (≡Si–OH) of partially dehydroxylated silica with a metal-alkyl to obtain in principle well defined grafted complexes.^2,9,15,16^ However, it appeared progressively that depending on the degree of dehydroxylation of a support, mono, bi and multipodal species can be generated.^2,9,17–19^

**Scheme 1:**
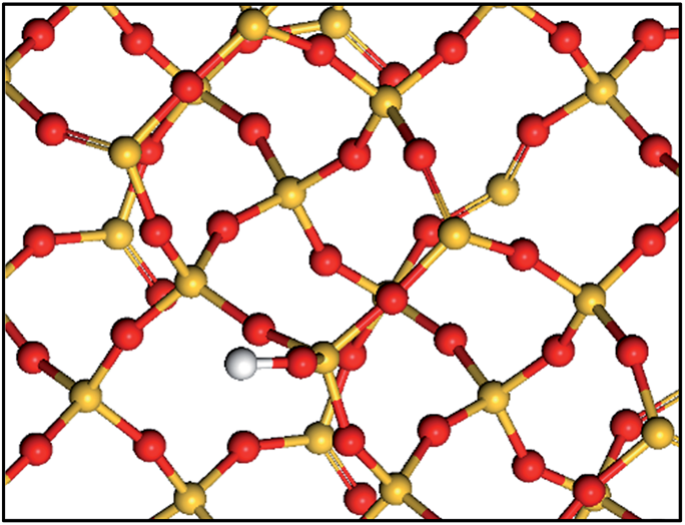
Simple representation of a partially dehydroxylated silica surface: isolated silanols and siloxane bridges. Si atoms are in yellow, O atoms are in red and H atoms are in white.

In fact there is growing evidence that the grafted complexes interact with the surrounding atoms present on the surface (Scheme 1).^2,9,20^

Indeed, the silica dehydroxylation treatment leads to a highly strained reactive surface that may participate in anchoring reactions.^21–25^ The resulting surface organometallic chemistry became more associated to an “ensemble” of surfaces atoms that makes this chemistry increasingly difficult to fully understand, approaching to some extent the complexity of classical heterogeneous catalysis (the concept of ensemble effect was invented in heterogeneous catalysis by metals).^26–28^

We present here an exemplary case of this complexity by studying a well-known organometallic compound which presents the advantage of being monomeric in solution and which has a highly oxophilic metallic atom (Al) susceptible of making strong bond(s) with the silica surface. We demonstrate how the well-defined surface precursor can be transformed on the silica surface into a supported Al hydride *via* a mechanism showing the complexity of strained siloxane bridges.

The type of reaction between alkyl aluminum compounds (AlR_3_, R = Me,^29–34^ Et,^35^ iBu…) and silica surface still remains not elucidated despite its huge applications as a co-catalyst for olefins polymerization.^36–39^ Because many aluminum alkyls exist in solution as dimers or trimers, we have chosen triisobutylaluminum (TIBA) because it is a monomer,^40^ with the hope it will remain monomeric once it is grafted to the surface. A preliminary account of this chemistry was started on mesoporous silica SBA15_700_.^41^ At that time diethyl ether was chosen to stabilize the Al precursor because we wanted to avoid chemical interaction with the surrounding sites. Subsequently, several studies appeared on this subject with various aluminum alkyls grafted on SBA15_500_ and revealed a more complex behavior.^42^ This previous study involved the reaction of SBA15 surface, dehydroxylated at moderate temperature (500 °C) with an excess of TIBA (3 eq./≡SiOH). Applying their experimental conditions, Coperet *et al.* observed significant incorporation of Al in the bulk of SBA15_500_. Based on the available data, the authors concluded that, under their experimental conditions, there is remarkable incorporation of Al into the framework of SBA15.^35,42^

**Scheme 2:**
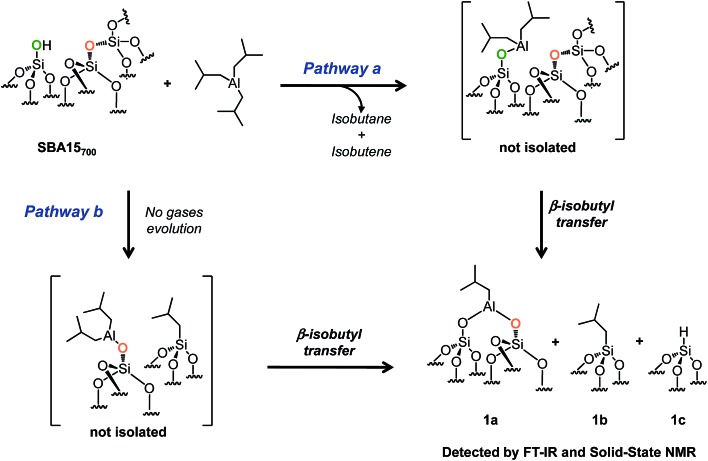
Reaction of TIBA (1 M/hexane) with single silanol (pathway a) and strained siloxane bridges (pathway b) of SBA_700_ at room temperature.

Facing this longstanding complex problem of the reactivity of TiBA with silica, we decided to re-investigate this reaction by a carefully chosen combination of the oxide support and experimental conditions. Here, a simple modification of the experimental conditions as compared to Coperet *et al.* works^42^ (1 : 1 ratio between TIBA and ≡SiOH and not 3 : 1) enables us to describe the stepwise interaction of TIBA with SBA15_700_ at room temperature avoiding thus the possible incorporation of Al in the bulk of SBA15. It occurs either by reaction with silanols (Scheme 2, pathway a) or by reaction with siloxane bridges ≡Si–O–Si≡ by two parallel pathways (Scheme 2, pathway b). Those materials were used as precursors of Al hydrides: a thermal treatment under vacuum of these grafted Al isobutyl leads, in the absence of hydrogen, to very stable Al hydrides that are tetra, penta or octahedral. Al hydrides^43^ have been known for almost a century as catalysts for the oligomerization of ethylene to linear α-olefins, and aluminum alkyls are used as cocatalysts in Ziegler–Natta polymerization of olefins.^44–50^

## Results and discussion

### Reaction of TIBA with SBA15_700_ surface

SBA15 is a well-known hexagonal ordered mesoporous support characterized by a high surface area (ESI†), large uniform pore diameter (6 nm), thicker walls (ranging from 3 to 6 nm) and high thermal stability (up to 1200 °C).^51,52^ After overnight dehydroxylation at 700 °C under high vacuum (10^–5^ mbar), the amount of isolated silanols (measured by titration with MeLi)^53^ is about 1.8 mmol g^–1^ (1.3 OH nm^–2^).

The reaction of 1 equivalent of TIBA (1 M/hexane)/silanol with SBA15_700_ leads to the formation of several compounds: [(≡SiO)_2_–Al–CH_2_CH(CH_3_)_2_] **1a**, silicon isobutyl [≡Si–CH_2_CH(CH_3_)_2_] **1b** and silicon hydride [≡Si–H] **1c** (Scheme 2). These results indicate that TIBA reacts not only with single silanols (Scheme 2, pathway a) but also with strained siloxane bridges (Scheme 2, pathway b).^41,54^ Interestingly – the amount of gas phase isobutane plus isobutene is close to the amount of grafted Al in principle in agreement with a protonolysis of one Al-isobutyl group and the microanalysis gives 8C/grafted Al (Tables S1 and S2, ESI†) – surprisingly the hydrolysis of the material gives only one mole of isobutane per grafted Al indicating that a ≡Si-isobutyl, which is not hydrolyzed, is already formed at room temperature simultaneously to Al-isobutyl.

**Fig. 1:**
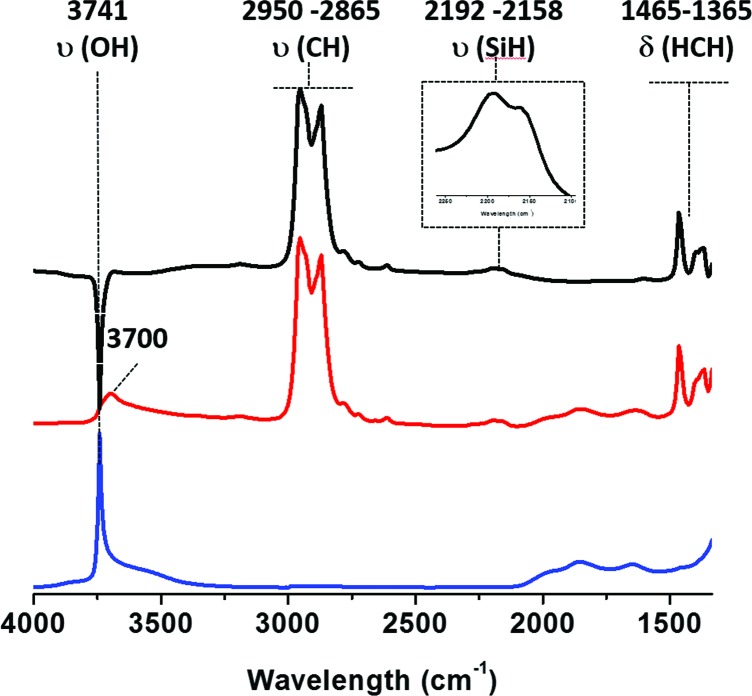
IR spectra of (i) SBA15_700_ (blue), (ii) SBA15_700_ after reaction with 1 equiv. of TIBA/≡SiOH (red) and (ii) – (i) subtraction of spectra (ii) and (i) (black).

The IR spectra obtained after reaction with TIBA (1 eq./≡SiOH) are shown in Fig. 1.

As expected, a partial consumption of the ≡Si–OH vibrational band is observed [3741 cm^–1^, *ν*(OH)]. Vibrational isobutyl groups appear at 2950 [*ν*_as_(CH_3_)], 2865 [*ν*_s_(CH_2_)], 1465 [*δ*_as_(CH_3_)] and 1365 cm^–1^ [*δ*_s_(CH_3_)]. Reaction of TIBA also led to the formation of silicon hydride(s) species characterized by a broad *ν*(Si–H) band at 2100–2200 cm^–1^.^39,55,56^ Based on DFT calculations (*vide infra*), the presence of these silicon hydrides is explained by a β-H elimination from one of the isobutyl fragment to the Al center of [≡SiO–Al–[CH_2_CH(CH_3_)_2_]_2_. Then, the Al–H intermediate thus formed reacts with adjacent silicon bridges to form silicon hydride.

The proton solid-state NMR spectrum of **1** displays five clear signals at 0.4, 0.9, 1.7, 2 and 4 ppm (Fig. S1, ESI†). According to both literature data and liquid state NMR of TIBA (Fig. S2 and S3, ESI†), the signals at 0.4, 0.9 and 2 ppm are assigned respectively to [(≡SiO)_2_–Al–C ***H***_**2**_CH(CH_3_)_2_], [(≡SiO)_2_–Al–CH_2_CH(C ***H***_**3**_)_2_] and [(≡SiO)_2_–Al–CH_2_C ***H*** (CH_3_)_2_]. The silicon hydride proton chemical shift appears at 4 ppm^55,57^ as expected from the FT-IR results.

Finally, the shoulder at 0.8 ppm is attributed to silicon isobutyl moiety [≡Si–C ***H***_**2**_CH(C ***H***_**3**_)_2_] which was suggested to be formed from analytical data.^58,59^ Another peak at 1.7 ppm is ascribed to unreacted silanols.

**Fig. 2:**
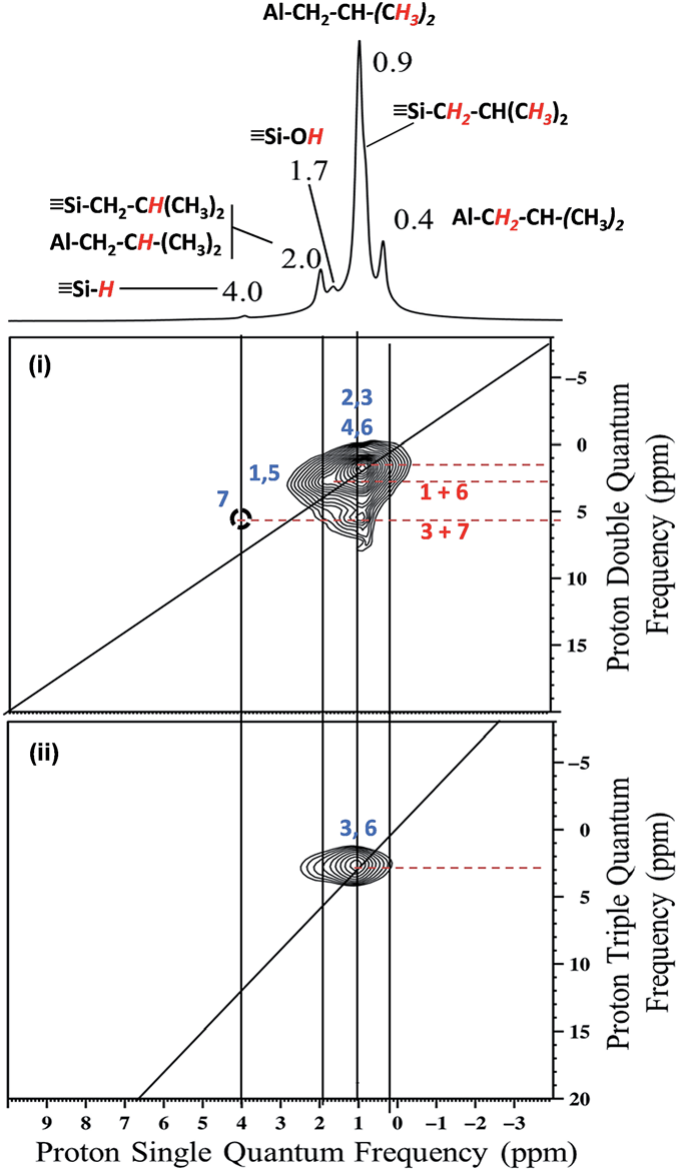
(i) DQ rotor-synchronized 2D ^1^H MAS NMR spectrum of **1**. (ii) ^1^H TQ MAS spectra of **1**.

The 2D double quantum (DQ) ^1^H-^1^H MAS NMR spectrum shows for the isobutyl group [C ***H***_**2**_CH(C ***H***_**3**_)]_2_ an intense autocorrelation on the 2 : 1 diagonal centered around 0.4–0.9 ppm in F2 and 0.8–1.8 ppm. Outside the diagonal, a strong correlation is observed at 2 ppm assigned to [≡Si–CH_2_C ***H*** (CH_3_)_2_] and [(≡SiO)_2_–Al–CH_2_C ***H*** (CH_3_)_2_] (Fig. 2i).

**Scheme 3:**
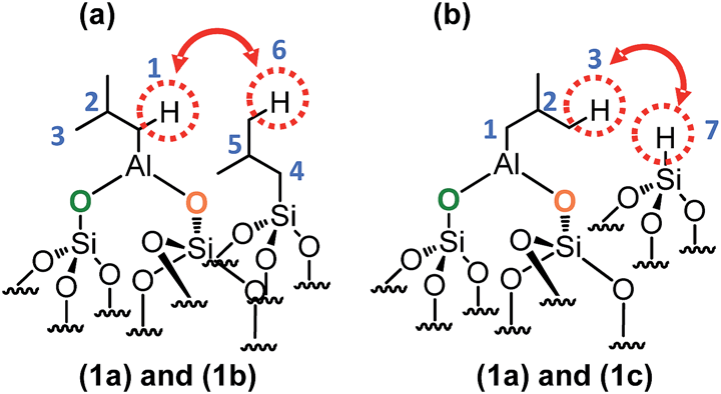
Representation of the close proximity of (a) aluminum isobutyl **1a** and silicon isobutyl **1b**, (b) aluminum isobutyl **1a** and silicon hydride **1c**.

This means that in the isobutyl fragments the ^1^H on the αC and βC are close together (Scheme 3a).

The proton resonance assigned to [≡Si–***H***] at 4 ppm shows a strong correlation with the proton at 0.9 ppm assigned to the methyl group (Fig. 2i). In other words, [≡Si–H] and [(≡SiO)_2_–Al–CH_2_CH(CH_3_)_2_] species are in close proximity (<5 Å) (Scheme 3b).

This suggests strongly that they were formed from the same elementary step on the surface.

The ^1^H triple quantum (TQ) solid state NMR spectrum of **1** highlights the expected correlation at 2.7 ppm corresponding to the proton of methyl group of [(≡SiO)_2_–Al–CH_2_CH(C ***H***_**3**_)_2_] and [≡Si–CH_2_CH(C**H**_**3**_)_2_] species, [3*δ_H_(CH_3_) = 3*0.9] (Fig. 2ii).

^13^C cross-polarization magic angle spinning (CP-MAS) spectrum shows a broad signal center at 24 ppm, assigned to Al–***C*** H_2_–CH–(CH_3_)_2_ and Si–CH_2_–***C*** H–(***C*** H_3_)_2_ associated with two shoulders at 23 and 26 ppm attributed to Si–***C*** H_2_– and Al–CH_2_–***C*** H–(***C*** H_3_)_2_, respectively (Fig. S4, ESI†).^41^ The ^29^Si NMR spectrum of **1** exhibits two signals centered at –106 ppm (broad) and –47 ppm (weak) (Fig. S5, ESI†). According to the literature, the former is attributed to both [(≡SiO)_3_SiOE], with E = Si or H (commonly called Q^4^ and Q^3^) and Si(OAl) (OSi)_3_ ^60^ and the latter is assigned to [(≡SiO)_2_Si(OH)X], with X = H or R (commonly called T^2^ site).^55^ Interestingly, the 1 : 1 ratio between TIBA/≡SiOH used in this work induces no significant Al incorporation in network as compared to Coperet *et al.* works.^42^ Indeed, no signal characteristic of Si(OAl)_4_ at – 85 ppm is observed.^60^

Finally, we performed ^27^Al NMR spectroscopy. This technique is usually an efficient probe to demonstrate the presence of alkyl group and to assess the coordinance of Al-alkyl center.^40,61^ The ^27^Al NMR spectrum of material **1** shows three clear signals at 5, 33 and 59 ppm (Fig. S6, ESI†) typically assigned to octahedral (Al_Oh_), pentahedral (Al_Ph_) and tetrahedral (Al_Th_),^62–65^ but not characteristic of alkyl complexes. Indeed, a tetrahedral Al-alkyl features a chemical shift of 117 ppm and a quadrupolar coupling constant of 17.5 MHz.^66^ However, as previously describes by Espinas *et al.* ^27^Al NMR signals are notoriously difficult to observe to very broad line shape.^41^ As frequently observed in SOMC,^2^ Al center coordinate to the silica surface (≡Si–O–Si≡) affording Al-surfaces complexes with different Al chemical shifts. The Al_Oh_ species are closer to the surface and so are assumed to be due to the reaction of TIBA with the strained siloxane whereas the Al_Th_ species are issued from the reaction of single silanol with TIBA. Al_Ph_ is probably the result of both reactions. Thus, material **1** displays three types of isobutyl aluminum geometries along isobutyl silicon and silicon hydride. To have a more detailed understanding of the reactivity of TIBA with SBA15_700_ a mechanistic rationalization of these results will be provided in the following section.

### Density functional theory calculations

The experimental results described so far can be explained with a variety of mechanisms. To achieve a more structured understanding of these systems we performed DFT calculations. We first report on the reactivity of TIBA with a siloxane bridge on the silica surface, then we move to analyze the reactivity of TIBA with a silanol protruding from the silica surface, and we continue with conversion of monopodal [≡SiO–Al–[CH_2_CH(CH_3_)_2_]_2_ into bipodal [(≡SiO)_2_–Al–CH_2_CH(CH_3_)_2_].

### Reaction of ≡Si–O–Si≡ with TIBA

**Fig. 3:**
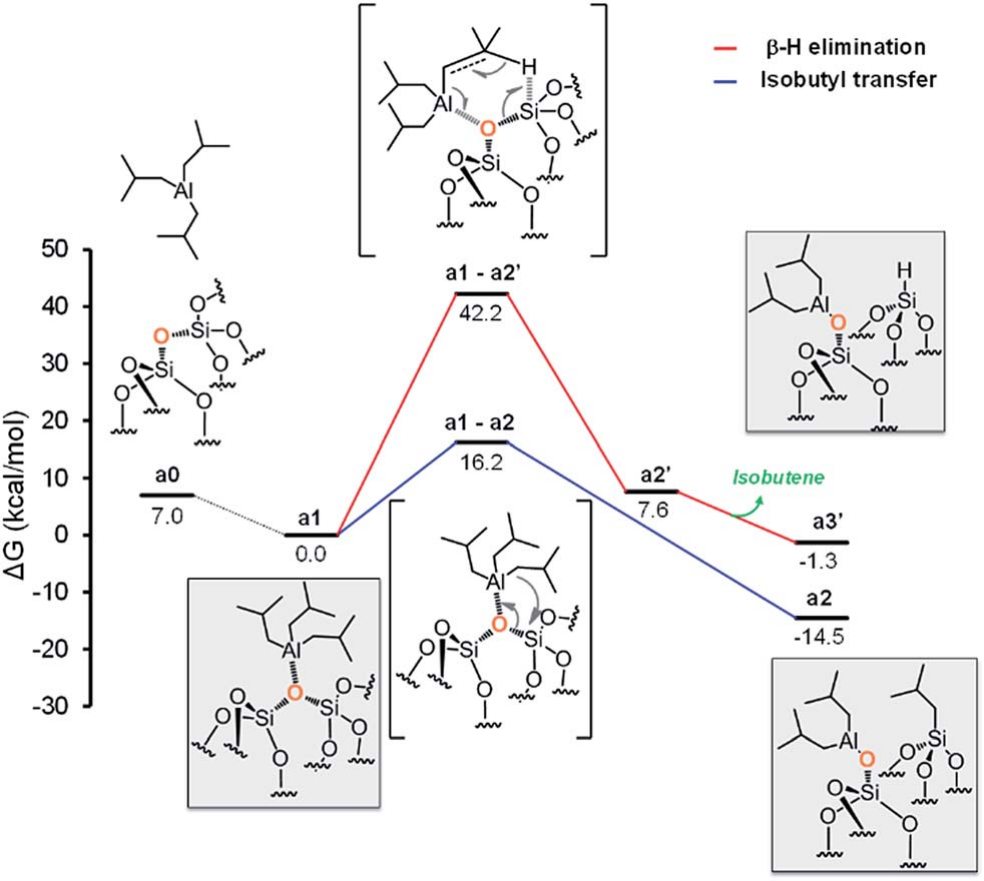
Energy profile for the reactivity of TIBA with siloxane bridges (≡Si–O–Si≡).

The initial step is coordination of the oxophilic Al center of TIBA to an O atom on the silica surface, to give intermediate **a1** with release of 7.0 kcal mol^–1^ (Fig. 3). For the sake of simplicity, in the following we will assume intermediate **a1** as reference structure at 0 kcal mol^–1^ in energy. Reactivity of **a1** can involve either the transfer of one of the isobutyl groups of TIBA to a Si atom, or the transfer of a β-H atom from one of the isobutyl groups of TIBA to a Si atom, with release of isobutene. The alkyl transfer pathway (blue profile) occurs through the four centers transition state **a1-a2**, 16.2 kcal mol^–1^ above **a1**. Product **a2**, presenting the [≡SiO–Al–[CH_2_CH(CH_3_)_2_]_2_] and [≡Si–CH_2_CH(CH_3_)_2_] moieties, lies 14.5 kcal mol^–1^ below the initial adduct **a1**. The β-H transfer pathway (red profile) occurs through the six centers transition state **a1-a2′**, 42.2 kcal mol^–1^ above **a1**, and collapses into the Al-coordinated isobutene intermediate **a2′**, 7.6 kcal mol^–1^ above **a1**.

Finally, isobutene dissociation leads to species **a3′**. Comparison of the two pathways of Fig. 3 clearly indicates that the only viable mechanism corresponds to alkyl transfer to form **a2**.

At this point, we should model reactivity of the formed monopodal [≡SiO–Al–[CH_2_CH(CH_3_)_2_]_2_] with a nearby siloxane bridge to yield bipodal [(≡SiO)_2_–Al–CH_2_CH(CH_3_)_2_]. However, since this conversion can also occur with a monopodal Al species deriving from initial reactivity of TIBA with a silanol (Scheme 2), we decided to use a general model for the monopodal Al to bipodal Al conversion, see the next section.

### Reaction of ≡Si–OH with TIBA

**Fig. 4:**
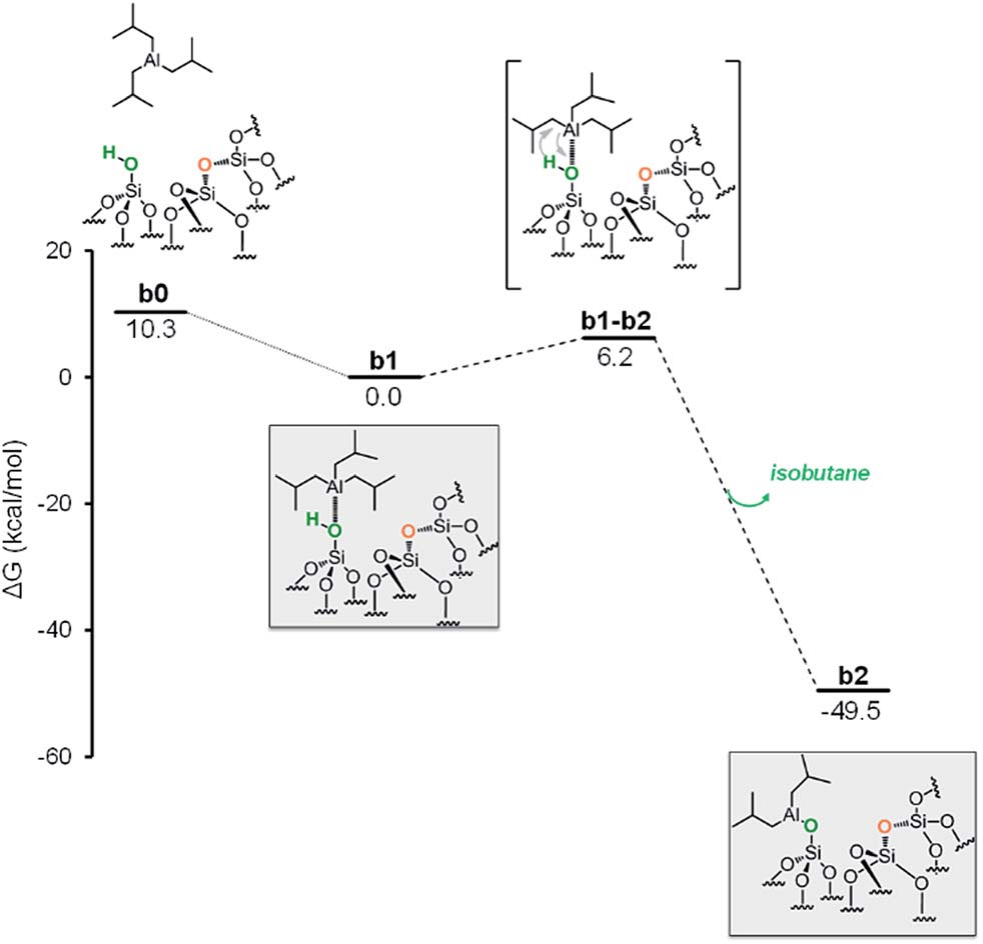
Energy profile for the reactivity of TIBA with isolated silanol (≡Si–OH).

The first step is the coordination of TIBA to the silanol, resulting in the intermediate **b1**, with an energy gain of 10.3 kcal mol^–1^ (Fig. 4).

The reaction occurs *via* σ-bond metathesis with release of isobutane through the four centers transition state **b1-b2**, 6.2 kcal mol^–1^ above **b1**. This leads to the monopodal [≡SiO–Al–[CH_2_CH(CH_3_)_2_]_2_] **b2**, 49.5 kcal mol^–1^ below **b1**.

**Fig. 5:**
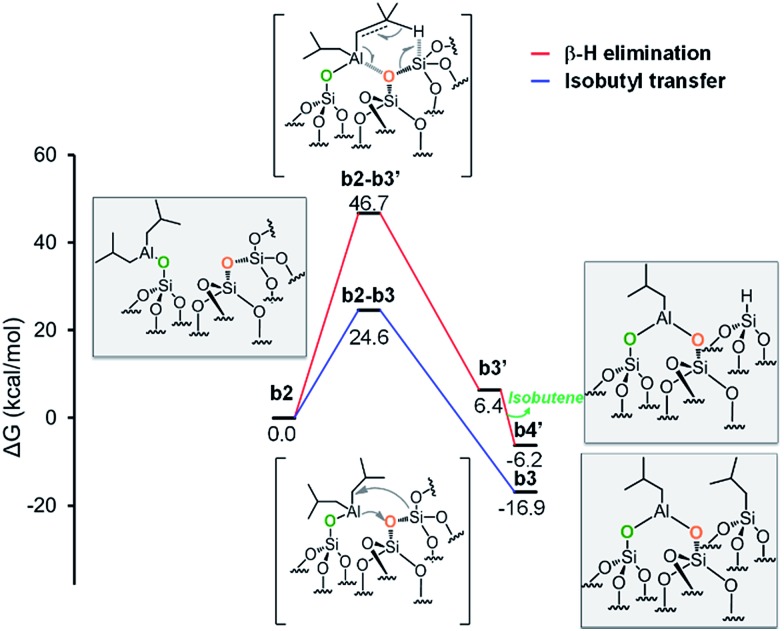
Energy profile for the reactivity of monopodal [≡SiO–Al–[CH_2_CH(CH_3_)_2_]_2_] with siloxane bridges, (≡Si–O–Si≡). Alkyl transfer *versus* direct H transfer.

As the starting point of the second step corresponds to the monopodal structure, in the following, we will assume intermediate **b2** as reference structure at 0 kcal mol^–1^ in energy. We envisaged three possible evolutions for intermediate **b2**. The first corresponds to the transfer of an isobutyl group from **b2** to a siloxane bridge on the surface, with formation of bipodal [(≡SiO)_2_–Al–CH_2_CH(CH_3_)_2_] and [≡Si–CH_2_CH(CH_3_)_2_], **b3** (Fig. 5, Scheme S1, ESI†). The second corresponds to the transfer of a β-H atom from one isobutyl group of **b2** to a siloxane bridge, with formation of bipodal [(≡SiO)_2_–Al–CH_2_CH(CH_3_)_2_] and of silicon hydride [Si–H], **b5′′** associated with the release of isobutene (Fig. 5, Scheme S2, ESI†). The third pathway starts with a β-H elimination from one of the isobutyl to the Al center, followed by the transfer of this H atom from Al to a siloxane bridge.

Focusing on the first pathway, the alkyl transfers occurs through the four centers transition state **b2-b3**, with a barrier of 24.6 kcal mol^–1^ from **b2** (Fig. 5, blue profile).

**Fig. 6:**
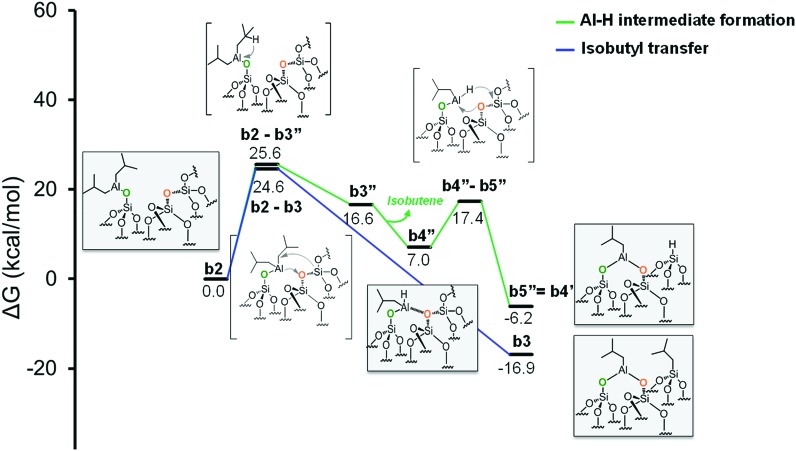
Energy profile for the reactivity of monopodal [≡SiO–Al–[CH_2_CH(CH_3_)_2_]_2_] with siloxane bridges, (≡Si–O–Si≡). Alkyl transfer *versus* two step H transfer.

Moving to the second pathway, the β-H transfer to the Si with elimination of isobutene occurs through the six centers transition state **b2-b3′**, with a barrier of 46.7 kcal mol^–1^ from **b2**. This result excludes this pathway from a possible reaction scenario (Fig. 5, red profile). The third pathway starts with a β-H elimination from one of the isobutyl to the Al center *via* transition state **b2-b3′′**, 25.6 kcal mol^–1^ above **b2**, and formation of intermediate **b3′′**. This intermediate presents an Al–H bond and an isobutene molecule still coordinated to the Al center. The dissociation of isobutene leads to intermediate **b4′′**, which presents an interaction between the Al center and an O atom of a nearby siloxane bridge. The transfer of the H center to the Si atom of the siloxane bridge occurs through the four centers transition state **b4′′-b5′′**, with a barrier of 10.4 kcal mol^–1^ from **b4′′**. The final product **b5′′** presents the bipodal [(≡SiO)_2_–Al–CH_2_CH(CH_3_)_2_] and [≡Si–H] (Fig. 6, green profile and Scheme S3, ESI†).

**Fig. 7:**
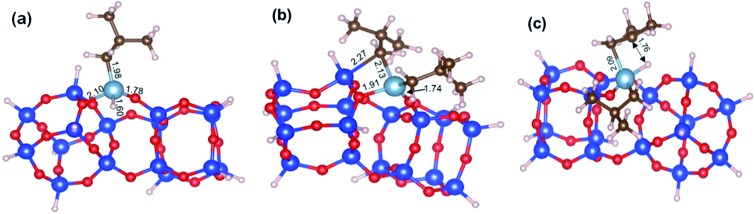
DFT optimized geometry of (a) the Al–H intermediate **b3′′**, (b) of the transition states **b2-b3** and **b2-b3′′**, for alkyl transfer to the silica, and (c) the β-H elimination to the Al. Distances are in Å.

While our results should be taken *cum grano salis*, since the exact energetics of the various transformations certainly depends on the specific model we used for the underlying silica surface, we believe they allow depicting a well-grounded scenario. First, alkyl transfer from **b2** to a siloxane bridge is clearly favored over β-H transfer with isobutene release, both kinetically and thermodynamically. Secondly, the barrier for the alkyl transfer to a siloxane bridge is of moderately low energy even with the poorly strained silica models we used. Third, reactivity of TIBA with the silanol is easier than reactivity of TIBA with a siloxane bridge, which is in agreement with the experiments discussed above. Fourth, formation of Si–H bonds can be only explained by a two-step mechanism, with initial β-H elimination to the Al, and formation of an Al–H bond (Fig. 7a), followed by H-transfer to a siloxane bridge. This reaction is in competition with the alkyl-transfer, selectivity being determined at the level of transition states **b2-b3** and **b2-b3′′**. With the specific model for silica we used, the alkyl transfer transition state **b2-b3** is favored by only 1 kcal mol^–1^ over the β-H elimination transition state **b2-b3′′**. Decomposition of this small free energy difference into electronic and thermal (including zero-point energy, ZPE, correction) contributions indicate that transition state **b2-b3** is favored by 9.5 kcal mol^–1^ in terms of electronic energy, and it is the thermal plus ZPE terms that reduce remarkably the free energy gap between the two transition states. This effect can be rationalized considering that transition state **b2-b3** is highly ordered, with the Al atoms strongly engaged with a siloxane bridge, which reduces remarkably the flexibility of the Al moiety, (Fig. 7b). Conversely, the β-H elimination transition state **b2-b3′′** is much more flexible, since the elimination occurs within the Al moiety, without any interaction with the silica surface, (Fig. 7c). This is well indicated by the presence of three vibrational modes of very low frequency (below 20 cm^–1^) in transition state **b2-b3′′**, corresponds to librational movements of the Al moiety. Despite the scarce accuracy of these low frequency vibrational modes within the harmonic approximation, which affects the exact value of the free energy gap between transition states **b2-b3** and **b2-b3′′**, they clearly provide an explanation for the competition between alkyl and β-H transfer to the silica surface.

Furthermore, DFT calculations are in qualitative agreement with the experimental data obtained from elemental and gas phase analysis. Indeed, quantification of both processes: alkyl transfer *vs.* two step H transfer (Fig. 6) is experimentally possible. The total amount of Al atoms in the surface is 1.35 mmol g^–1^ (Table S1†). The amount of isobutene, coming from the generation of Si–H *via* Al–H intermediate and due to the two step H transfer, is 0.5 mmol g^–1^ (Table S2†). This two step H transfer process corresponds to the ratio between the amount of isobutene and the amount of total Al. Finally, the two step H transfer occurs at 37% and the alkyl transfer at 63%. The latter is favored according to both experimental and DFT calculations.

### Generation of aluminum hydrides species

**Scheme 4:**
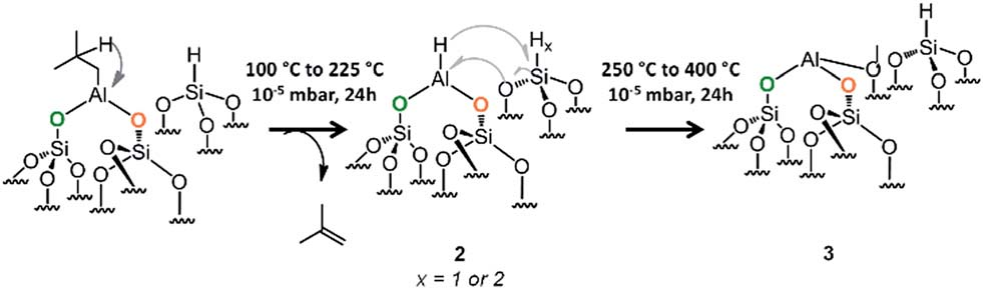
Formation of aluminum hydride through a thermal treatment (100 °C to 400 °C) under 10^–5^ mbar for 20 h of 1.

Aluminum hydride have been already formed on γ-alumina but the process required harsh temperature conditions (400 °C) under H_2_ pressure of 0.733 bar.^43^ We were interested in the formation of aluminum hydride species through a variable temperature treatment (100 °C to 400 °C, rate 60 °C h^–1^, Fig. S8 and S9, ESI†) under high vacuum (10^–5^ mbar). This process was followed by FT-IR spectroscopy and the best temperature to achieve the formation of aluminum hydride is 225 °C (Fig. S9 and S10,† Scheme 4).

At 225 °C, the FT-IR spectrum shows a strong decrease of the vibrational bands of isobutyl groups at 2950 [*ν*_as_(CH_3_)], 2865 [*ν*_s_(CH_2_)], 1465 [*δ*_as_(CH_3_)] and 1365 cm^–1^ [*δ*_s_(CH_3_)].

**Fig. 8:**
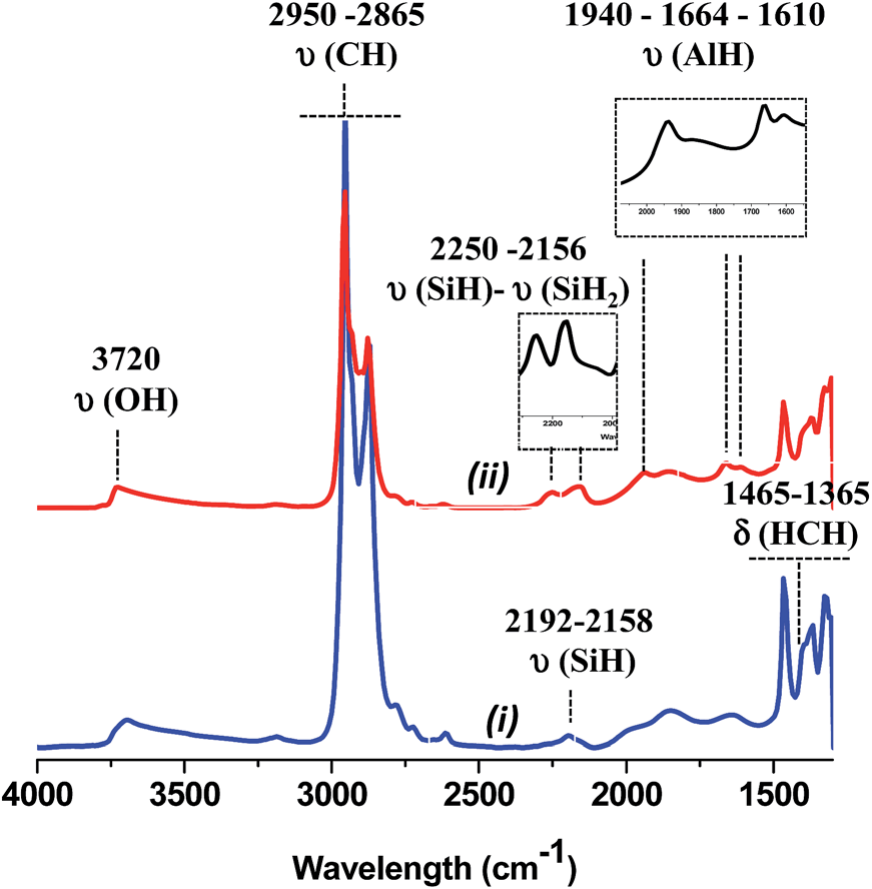
(i) IR spectra of **1**, (ii) after thermal treatment at 225 °C for 24 h. Inlet subtraction (ii) – (i) in the corresponding area of silicon and aluminum hydride, respectively.

Besides, three new bands appear at 1940, 1664 and 1610 cm^–1^ (Fig. 8).^43,67^ They are easily attributed to the vibration bands of Al–H. Indeed, terminal Al–H elongation bands range down to 1800 cm^–1^, bridging Al–H–Al give rise to signals between 1750 and 1550 cm^–1^.^67^ Thus, the β-H elimination occurs from Al-isobutyl and results on the formation of Al–H (terminal and bridging) characterized by vibration bands at 1940 and 1664, 1610 cm^–1^, respectively. Note, that a hydride transfer from Al–H to closer siloxane could also occur with the temperature to form either silicon hydride or bis-hydride characterized by two *ν*(Si–H) band at 2250 and 2156 cm^–1^, respectively. At 300 °C, the vibration band assigned to Al–H decreases significantly upon the increase of the characteristic vibration band of silicon hydride. Above 300 °C, no Al–H remains, only the silicon monohydride species are present.^68^ But it is interesting to mention that the aluminum hydride species formed at 225 °C are highly stable with time at room temperature under static vacuum (Fig. S10, ESI†).

**Fig. 9:**
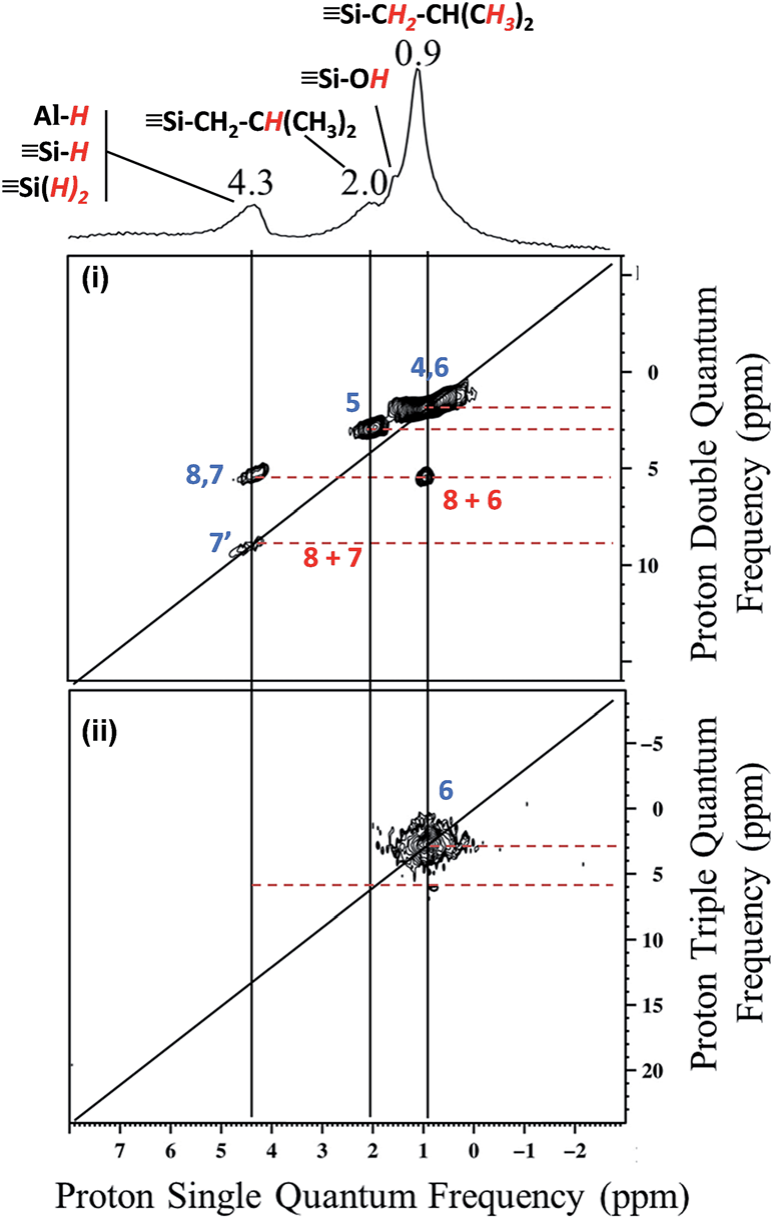
(i) DQ rotor-synchronized 2D ^1^H MAS NMR spectrum of **2**. (ii) ^1^H TQ MAS spectra of **2** (7′ is ascribed for SiH_2_).

To reinforce these results we performed solid state NMR spectroscopies of sample treated at 225 °C for 24 h under dynamic vacuum (Fig. 9).

The ^1^H NMR spectrum of **2** shows the disappearance of the signal characteristic of [(≡SiO)_2_–Al–C ***H***_**2**_CH(CH_3_)_2_] species at 0.4 ppm. Only the silicon isobutyl remains on the surface, as the peaks at 0.9 and 2 ppm are still present. It is in accordance with the ^13^C CP-MAS NMR spectrum where only the chemical shift at 24 ppm is observed (Fig. S11, ESI†). The signal at 26 ppm assigned to Al–CH_2_–***C*** H–(***C*** H_3_)_2_ is no longer detected. The broad ^1^H signal at 4.3 ppm is attributed to Al–***H***,^43^ Si–***H*** and Si ***H***_**2**_.^55,56,68^

**Scheme 5:**
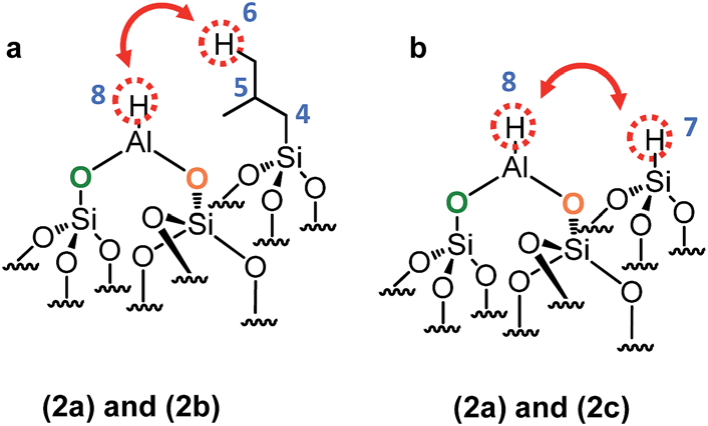
Representation of the close proximity of (a) aluminum hydride **2a** and silicon isobutyl **2b**, (b) aluminum hydride **2a** and silicon hydride **2c**.

In the 2D ^1^H-^1^H and in TQ spectra (Fig. 9), a strong auto-correlation is observed at 0.9 ppm. It corresponds to the proton signal of the [≡Si–C**H**_**2**_–CH (C**H**_3_)_2_]. Besides in the spectrum of Fig. 9i, a strong correlation is observed between the proton of Al–***H*** and the [≡Si–CH_2_–CH(C ***H***_**3**_)_2_]. In other words, Al–H and silicon isobutyl are in close proximity (<5 Å) (Scheme 5a).

**Fig. 10:**
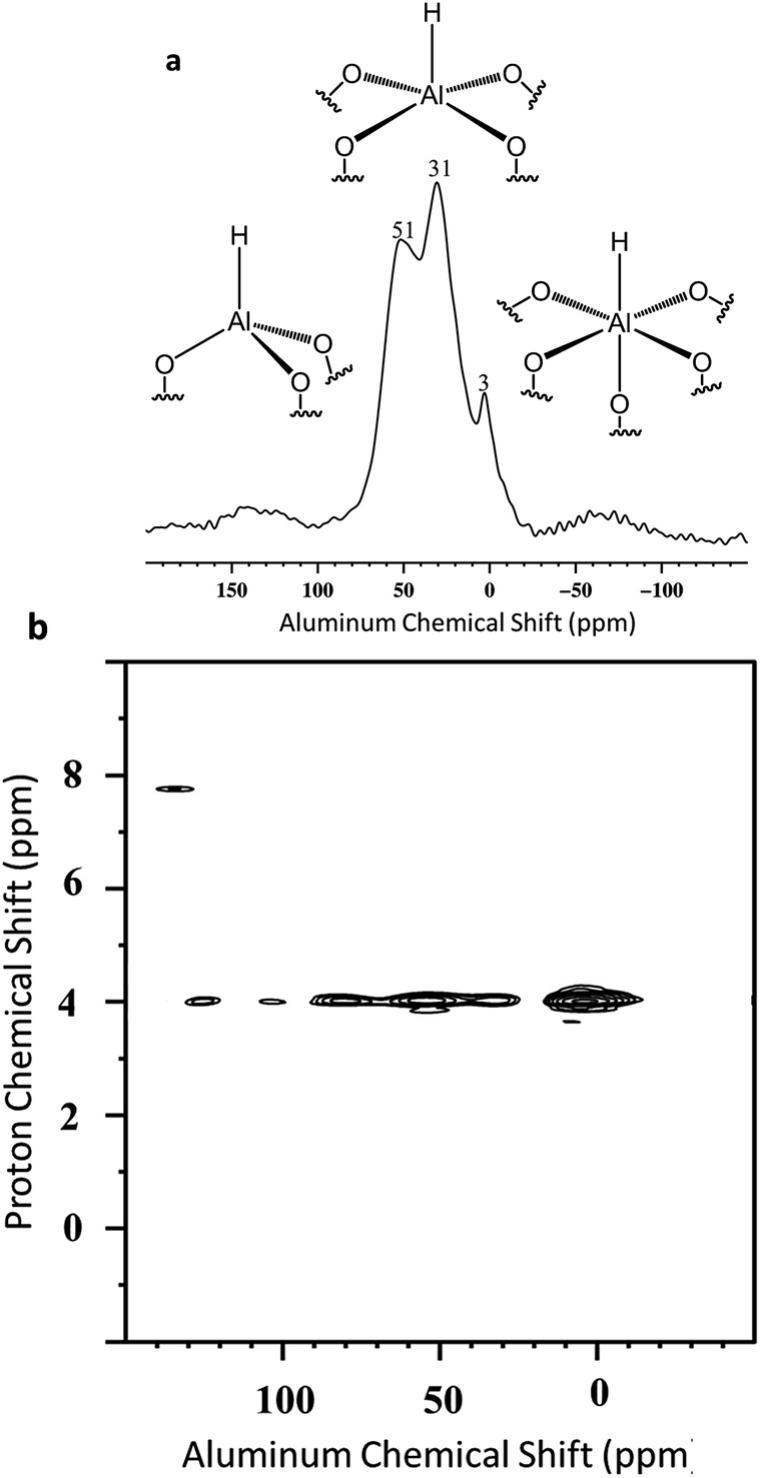
(a) ^27^Al MAS solid-state NMR spectrum of **2** and (b) ^27^Al–^1^H J-HMQC MAS NMR spectrum of **2**.

Besides, Al–H and Si–H are also close (Scheme 5b) due to the correlation centered at 8.6 ppm [*δ*_H_(Al–***H***) + [*δ*_H_(Si–***H***) = 4.3 + 4.3]. Finally to confirm the presence of the different coordination mode of the Al–H species, ^27^Al solid state NMR was performed (Fig. 10a). As expected and in accordance with the FT-IR studies, Al_Th/Ph_ hydride species are assumed to be present in more important proportion than Al_Oh_ hydride species. The latter one reacts with closer siloxane bridge to form silicon hydride species. The ^27^Al–^1^H correlation spectrum in Fig. 10b was acquired using the J-HMQC pulse sequence which correlates nuclei through their scalar coupling, a method well suited for hydride species presenting large ^1^J_Al–H_ couplings. The broad signal along the ^27^Al dimension corresponds to only those aluminum centers bearing hydride ligands. Thus, the proton signal at 4.3 ppm correlates with the ^27^Al signals at 3, 31 and 51 ppm corresponding respectively to octa, penta and tetrahedral aluminum hydride.

The spectrum of Fig. 10b clearly shows that Al_Oh_ are more abundant than Al_Th/Ph_. Interestingly, the spectrum of Fig. 10a, reveals the opposite: few Al_Oh_ at 3 ppm. Thus, the projection of the 2D ^1^H–^27^Al J-HMQC MAS NMR spectrum in the ^27^Al dimension was performed to provide some semi-quantitative information regarding the coordinance of supported Al–H (Fig. S12†). These data confirm the predominance of Al_Th/Ph_ towards Al_Oh_. Finally, the J-HMQC-filtered ^1^H MAS spectrum (Fig. S13†) shows the presence of a ^1^H site at a chemical shift of 4 ppm, in a major proportion. This chemical shift lies above the expected range for molecular aluminum hydrides.^69^

## Conclusions

The mechanism of reaction of TIBA with SBA15_700_ is clearly elucidated by combining experimental data and DFT calculations. Both show the formation of a monopodal bis-isobutyl aluminum intermediate due to simultaneous protonolysis and opening siloxane bridge. This intermediate leads to a bipodal bissiloxy isobutyl aluminum involving the formation of either a silicon isobutyl (alkyl transfer) or a silicon hydride (β-H elimination *via* aluminum hydride intermediate). From this material **1**, we are able to generate aluminum hydride **2** through a thermal treatment at 225 °C under high vacuum. This is possible by a β-H elimination. All these supported aluminum materials were fully characterized by FT-IR and advanced solid state NMR spectroscopies. The results are in accordance with the formation of tetra, penta and octahedral stable aluminum hydride. This study is a key step for the design of single site silica supported aluminum (alkyl or hydride).
