# Supplementary material for: Well-defined silica supported aluminum hydride: another step towards the utopian single site dream?
Source: Chem Sci. 2015 Jul 20;6(10):5456–65. doi: 10.1039/c5sc02276b (PMC5505125; doi:10.1039/c5sc02276b)
Supplement: Supplementary file 1 [file SC-006-C5SC02276B-s001.pdf]

Electronic Supplementary Material (ESI) for Chemical Science.
This journal is © The Royal Society of Chemistry 2015

Supporting Information

Well-Defined Silica Supported Aluminum Hydride: Another Step Towards the Utopian Single Site Dream?

Baraa Werghi, Anissa Bendjeriou-Sedjerari, Julien Sofack-Kreutzer, Abdesslem Jedidi, Edy Abou-Hamad, Luigi Cavallo*, Jean-Marie Basset*

jeanmarie.basset@kaust.edu.sa

luigi.cavallo@kaust.edu.sa

General procedure

All experiments were carried out under controlled atmosphere. Treatments of the surface species were carried out using high vacuum lines (1.34 Pa) and glove box techniques. Pentane was distilled on Na-benzophenone and degassed through freeze pump thaw cycles. Hydrogen was dried over freshly regenerated molecular sieves (3 Å) and deoxo traps.

Infrared spectra were recorded on a Nicolet 6700 FT-IR spectrometer equipped with a cell under controlled atmosphere.

Elemental analyses were performed at Mikroanalytisches Labor Pascher (Germany).

Nitrogen adsorption–desorption isotherms at 77 K were measured using a Micromeritics ASAP 2024 physisorption analyzer. Specific surface areas were calculated following typical BET procedures. Pore size distribution was obtained using BJH pore analysis applied to the desorption branch of the nitrogen adsorption/desorption isotherm.

Gas phase analysis of alkanes and alkene was performed on Agilent 6850 gas chromatography with split/splitless injector and FID. 10 µl was injected by the hot needle technique (thermospray) at an injector temperature of 250°C using the split mode (split ratio 50:1; 100 ml/min split flow). A HP-AL/KCl 50m × 0.32mm; 8.00 µm capillary column coated with a stationary phase divinylbenzene/ethylene glycol dimethacrylate was used with nitrogen as carrier gas at 18.3 Psi pressure. Each analysis was carried out with the same conditions: a flow rate of 2 ml/min, an isotherm at 80°C for 12 min then at 170°C for 3 min , and a detector sets with a data rate of 5 Hz and a minimum peak width of 0.04 min.

Solid State Nuclear Magnetic Resonance Spectroscopy:

One dimensional (1D) ^{1}H MAS, ^{13}C and ^{29}Si CP/MAS solid state NMR spectra were recorded on Bruker AVANCE III spectrometer operating at 600 MHz resonance frequency for ^{1}H utilized a 3.2 mm double resonance probe. ^{29}Si NMR solid state NMR was recorded using a 400 MHz Bruker AVANCE III spectrometer with a conventional double resonance 4 mm CP/MAS probe. In all cases the samples were packed into rotors under inert atmosphere inside glove boxes. Dry nitrogen gas was utilized for sample spinning to prevent degradation of the samples. NMR chemical shifts are reported with respect to the external references TMS and adamantane. For ^{13}C and ^{29}Si CP/MAS NMR experiments, the following sequence was used: 900 pulse on the proton (pulse length 2.4 s), then a cross-polarization step with a contact time of typically 2 ms, and finally acquisition of the ^{13}C and ^{29}Si NMR signal under high power proton decoupling. The delay between the scans was set to 4 s to allow the complete relaxation of the ^{1}H nuclei and the number of scans ranged between 3 000 - 25 000 for ^{13}C, 30 000 - 50 000 for ^{29}Si and was 8 for ^{1}H. An exponential apodization function corresponding to a line broadening of 80 Hz was applied prior to Fourier transformation.

^{1}H-^{1}H multiple-quantum spectroscopy: Two-dimensional double-quantum (DQ) and triple-quantum (TQ) experiments were recorded on a Bruker AVANCE III spectrometer operating at 600 MHz with a conventional double resonance 3.2 mm CP/MAS probe, according to the following general scheme: excitation of DQ coherences, t1 evolution, z-filter, and detection. The spectra were recorded in a rotor synchronized fashion in t1 by setting the t1 increment equal to one rotor period (45.45 µs). One cycle of the standard back-to-back (BABA) recoupling sequences was used for the excitation and reconversion period.[1-3] Quadrature detection in w1 was achieved using the States-TPPI method. An MAS frequency of 22 kHz was used. The 90° proton pulse length was 2.5 µs, while a recycle delay of 5 s was used. A total of 128 t1 increments with 128 scans per each increment were recorded. The DQ frequency in the w1 dimension corresponds to the sum of two single quantum (SQ) frequencies of the two coupled protons and correlates in the w2 dimension with the two corresponding proton resonances.2 The TQ frequency in the w1 dimension corresponds to the sum of the three SQ frequencies of the three coupled protons and correlates in the w2 dimension with the three individual proton resonances. Conversely, groups of less than three equivalent spins will not give rise to diagonal signals in this spectrum.

^{27}Al MAS and HMQC NMR:
The ^{27}Al NMR spectra were obtained from Bruker AVANCE III spectrometer operating at 900 MHz for ^{1}H with a conventional double resonance 3.2 mm CP/MAS probe with a recycling time of 1 s and a short pulse time 1 µs. The spinning frequency was 20 KHz and Al(H_2O)63+ was taken as a reference. The details of the experimental procedures for the 2D ^{27}Al HMQC NMR measurements were already described3. The spectra were obtained with Bruker AVANCE III spectrometer operating at 900 MHz for ^{1}H. Samples are spun at 20 KHz. The 2D H-1/Al correlation via heteronuclear zero and double quantum coherence with decoupling during acquisition. The recycling time was 1s with pulses of 3 and 6 µs.

Experimental procedure:

The reaction of SBA15 with TIBA was carried out in a double schlenk tube. 0.5 g of SBA15 was introduced with 1, 2.5 or 5 equivalents of 1M of TIBA in hexane were added and diluted in 3 ml of dry pentane.

The evolved gases were evacuated in a big 10L flask, and the white powder was washed with the dry pentane twice to eliminate the unreacted TIBA and left under vaccum for drying a whole night.

The C/Al ratio of 8.7 (1 equiv.), 8.8 (2.5 equiv.) and 8.9 (5 equiv.) are consistent with those expected (*th.* 8) for the formation of the species **1a** and **1b** in equimolar ratio (Table S1). Simultaneously, isobutane and isobutene are selectively evolved (Table S2). The isobutane/isobutene ratios depend on the amount of TIBA used. When 1 equiv. of TIBA/silanol is added, isobutane/isobutene ratio is 1.1 ± 0.2 are evolved. Increasing

the amount of TIBA from 2.5 to 5 equiv. leads to an increase of the isobutane/isobutene ratio from 2.0 to 3.2 respectively. Further proof of the composition of grafted material came from the hydrolysis of these compounds at room temperature that gives 0.80± 0.2 equiv. isobutane per Al whatever the amount of TIBA. Because hydrolysis are expected to cleave all the isobutyl groups linked to aluminum, the difference between the isobutane evolved by hydrolysis and the elemental analysis suggests that part of isobutyl is linked to a silicon atom (this is be confirmed by NMR), the other one being linked to Al. In parallel, the Al/isobutane ratios are not the one expected for a protonolysis of silanol. An increase of the reactant/silanols results in a decrease of the Al/*i*Butane ratio. In parallel, Al/isobutene ratios increase. These results indicate clearly that TIBA reacts not only with single silanol but also with what we believed to be a "hypothetically inert" strained siloxane bridges.

Table S1. Elemental analysis data[a]

TIBA/Si-OH (Calculated on the basis of molar equiv. of TIBA/≡SiOH present on SBA15 $_{700}$)	Al wt. % (mmol/g)	C wt. % (mmol/g)	C/Al ratio (th.)
1	3,66 (1.35)	14.07 (11.72)	8.68 (8)
2.5	4.52 (1.67)	17.6 (14.66)	8.77 (8)
5	5.2 (1.92)	20.69 (17.24)	8.94 (8)

[a] 0.05 to 0.1% error are estimated.

Table S2. Gas phase analysis data[b]

TIBA/Si-OH (calculated on the basis of molar equiv. of TIBA/≡SiOH present on SBA15 $_{700}$)	After grafting			After hydrolysis
	Al/Isobutane	Al/Isobutene	Isobutane/Isobutene	Isobutane/Al
**1**	2.7	3.3	1.1	0.8
**2.5**	1.75	3.6	2.075	-
**5**	1.43	5	3.2	0.89

[b] 10% of error is considered for the neopentane quantification by GC.

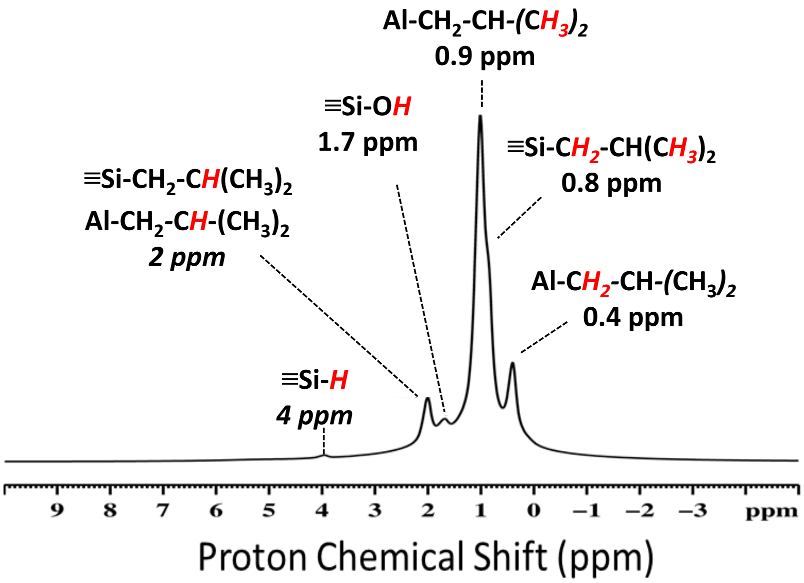

Figure S1: Solid-state ^{1}H CP-MAS NMR spectrum of 1 corresponding to the reaction of SBA15$_{700}$ with 1 equiv. of TIBA at RT for one hour.

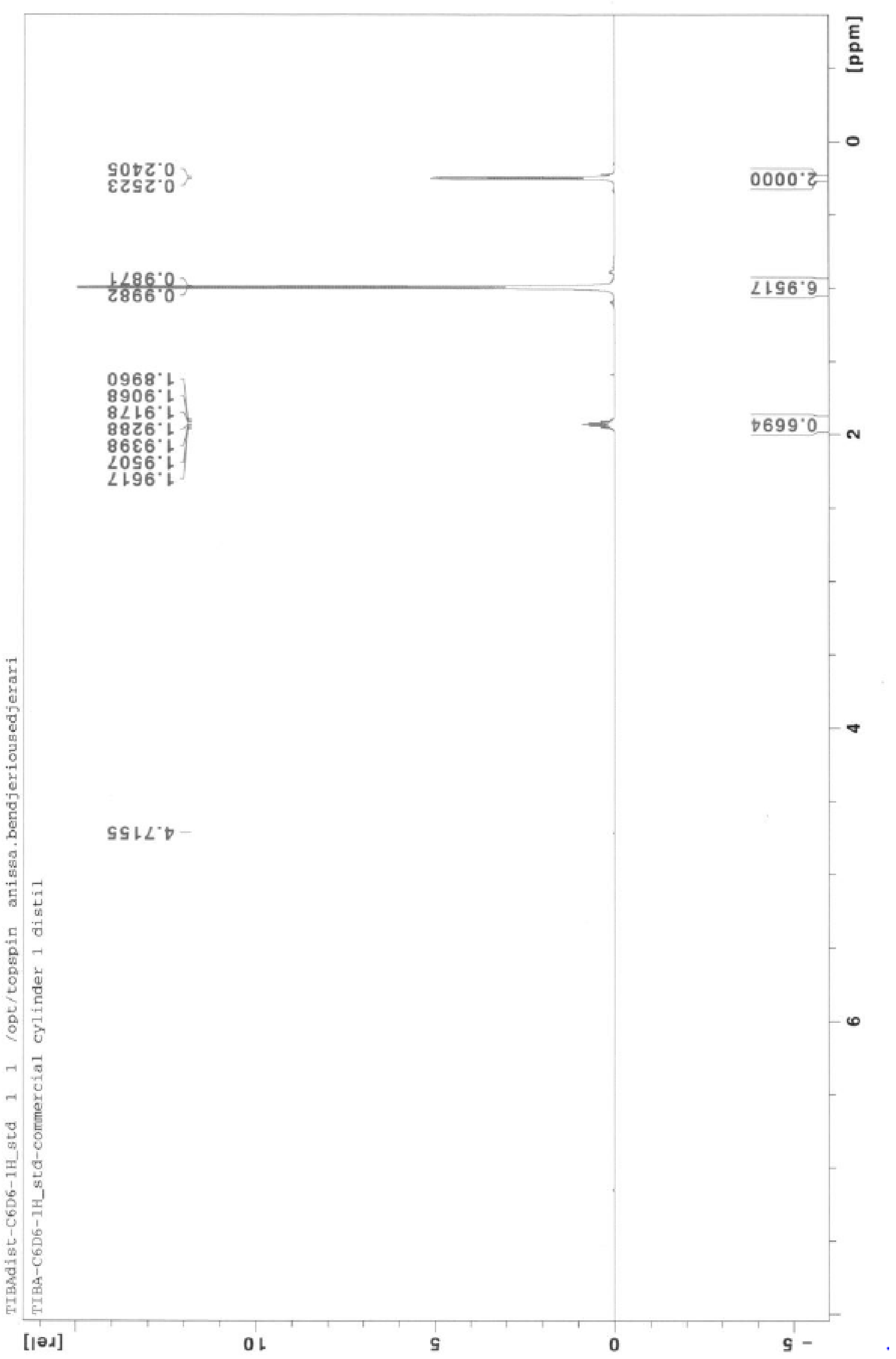

Figure S2: ^{1}H liquid NMR spectrum of TIBA in C_6D_6.

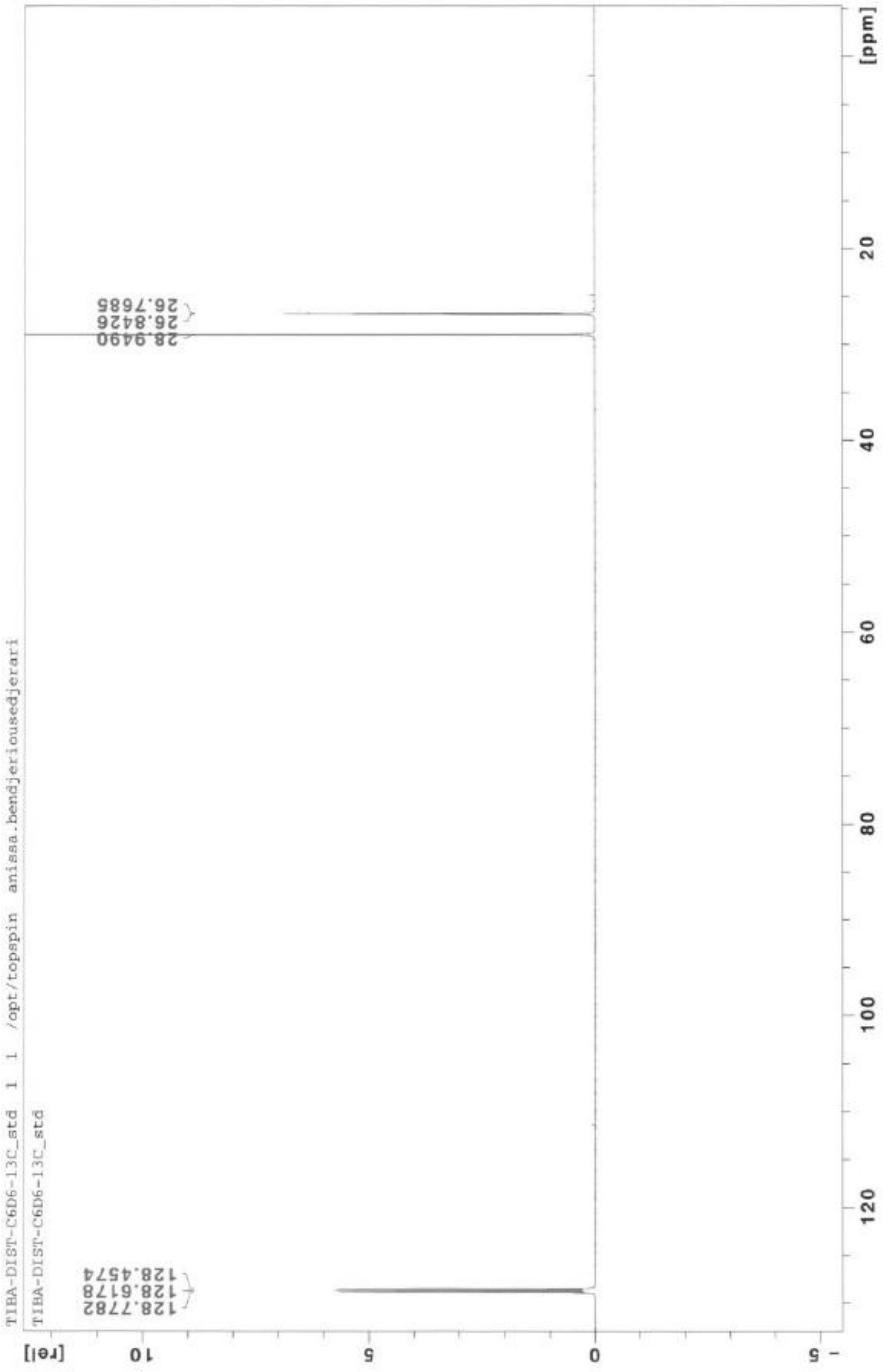

Figure S3: ^{13}C liquid NMR spectrum of TIBA in C_6D_6.

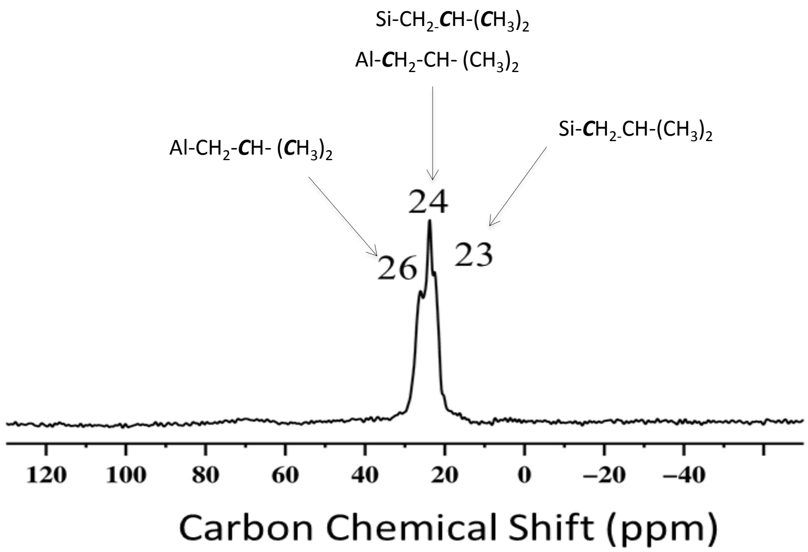

Figure S4: Solid-state ^{1}H-^{13}C CP-MAS NMR spectrum of 1 corresponding to the reaction of $SBA15_{700}$ with 1 equiv of TIBA at RT for one hour.

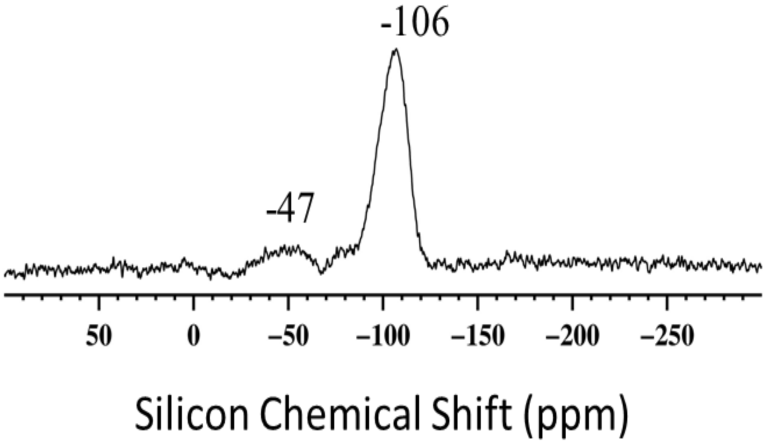

Figure S5: Solid-state ^{1}H-^{29}Si CP-MAS NMR spectrum of 1 corresponding to the reaction of $SBA15_{700}$ with 1 equiv of TIBA at RT for one hour.

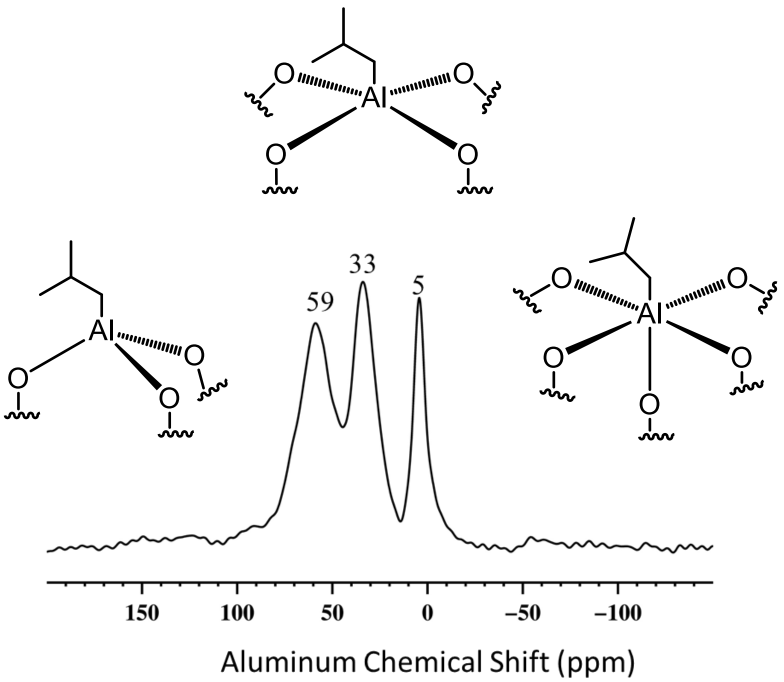

Figure S6: ^{27}Al MAS solid-state NMR spectrum of 1 corresponding to the reaction of SBA15$_{700}$ with 1 equiv of TIBA at RT for one hour.

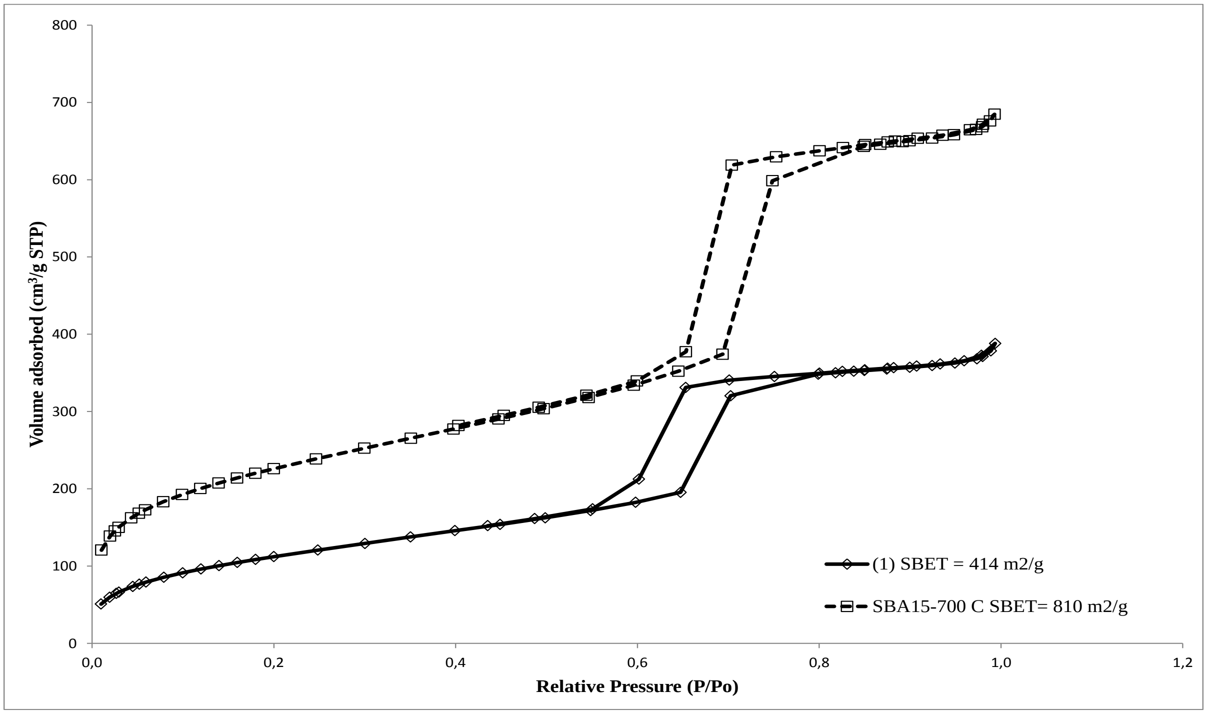

Figure S7: Nitrogen adsorption-desorption isotherms of of SBA-15$_{700}$ and 1.

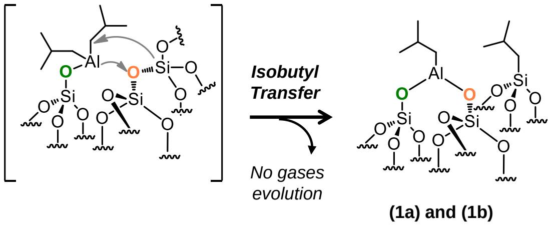

Scheme S1. A β-isobutyl transfer mechanism

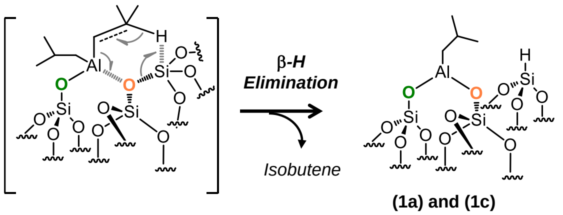

Scheme S2. Concerted β-H elimination via the formation of six-member ring transition state.

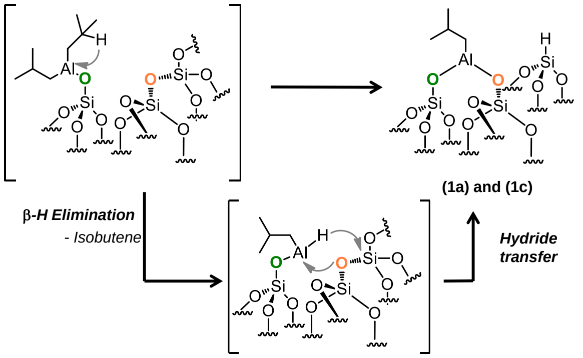

Scheme S3. β-H elimination via the formation of Al hydride intermediate.

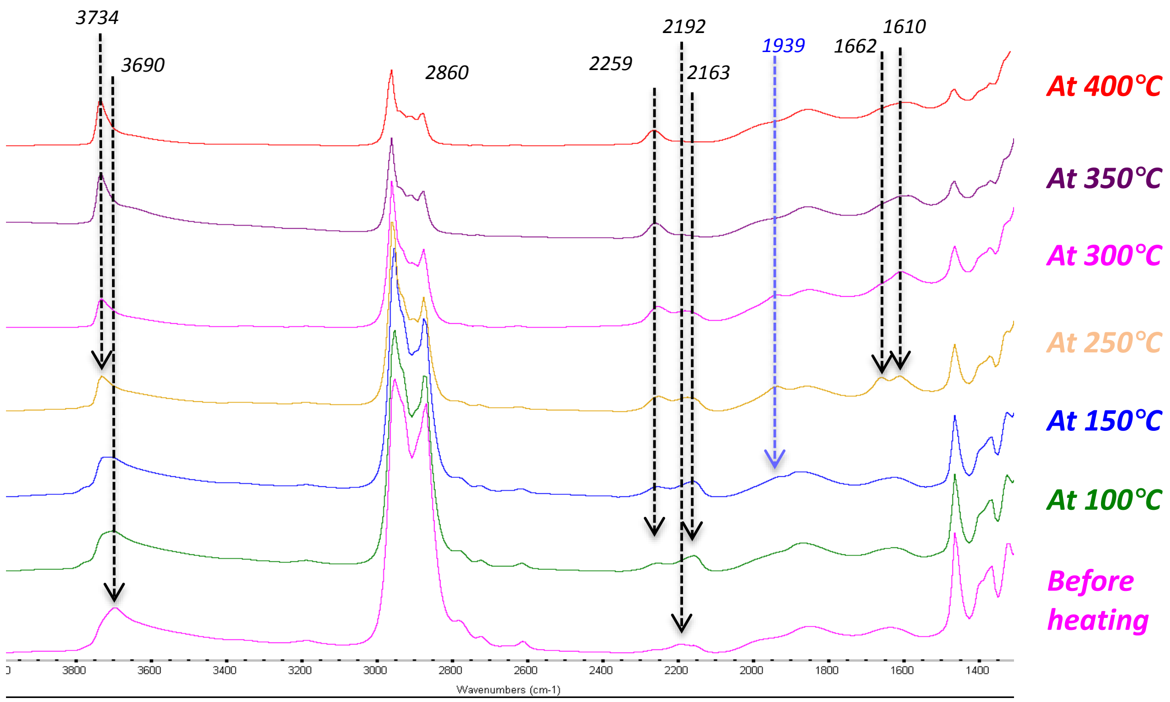

Figure S8: Variable temperature treatment of 1 from 100 °C to 400 °C (rate 60 °C / h) under high vacuum (10^{-5} mbar) following by FT-IR spectroscopy.

Assignments	IR bands (cm^{-1})
υ (OH)	3740
	3690 (Shifted due to the alkyl –OH interaction)
υ (CH)	2950-2860
υ (Si-H)	2192-2163
υ (Si-H2)	2259
υ (Al-H)	1939-1662-1610
δ (CH)	1460

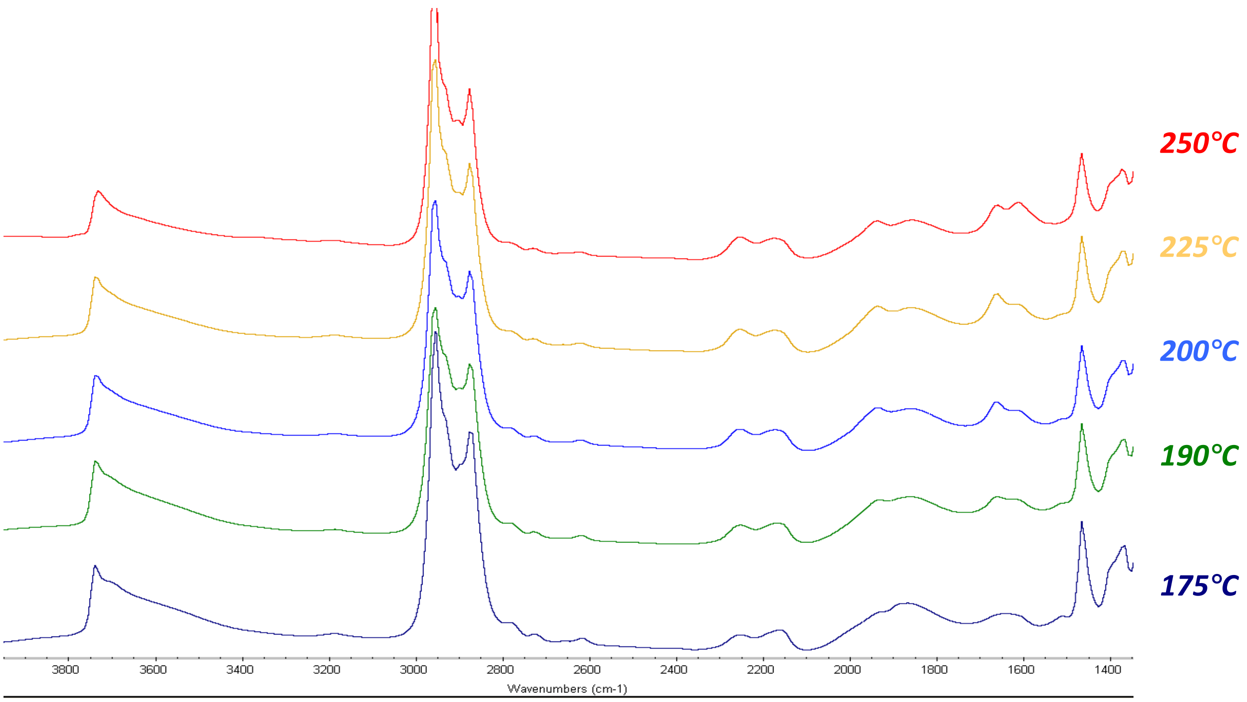

Figure S9: Variable temperature treatment of 1 from 175 °C to 250 °C (rate 60 °C / h) under high vacuum (10^{-5} mbar) following by FT-IR spectroscopy.

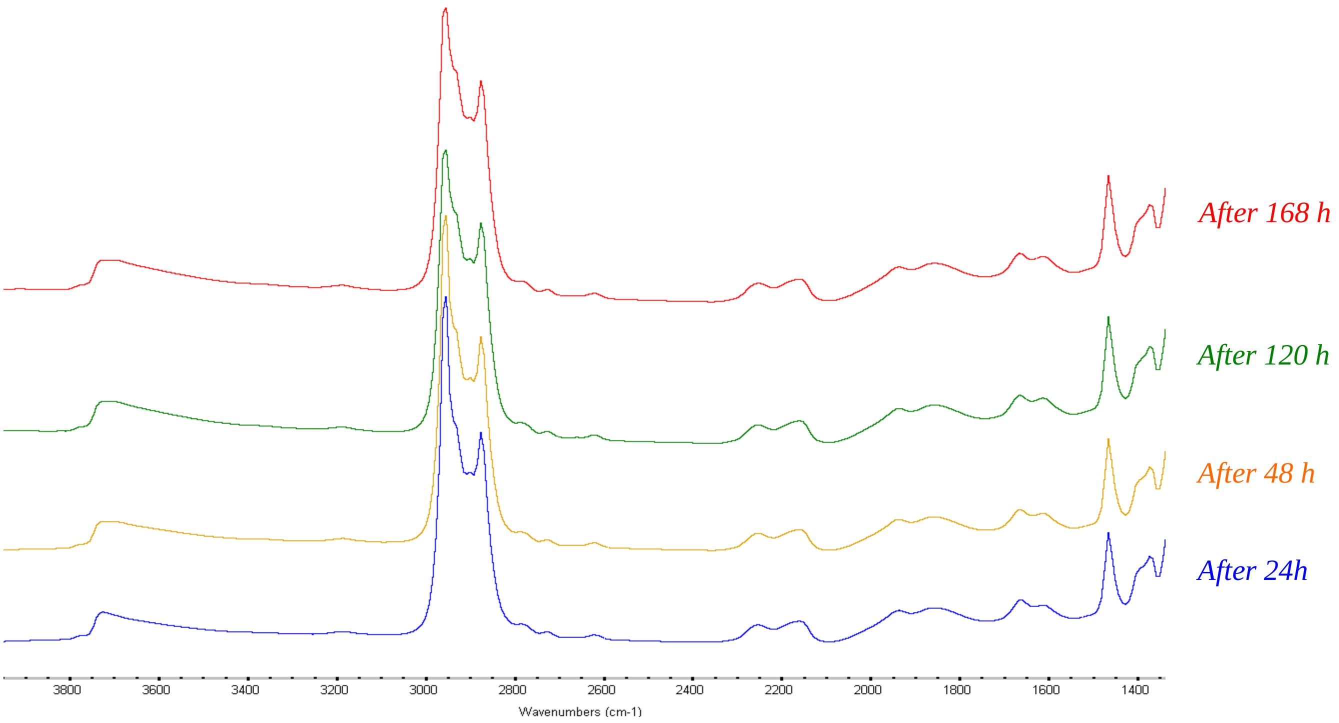

Figure S10: The FT-IR Study of the Al-H bond stability formed at 225°C with time at room temperature in a dynamic vaccum.

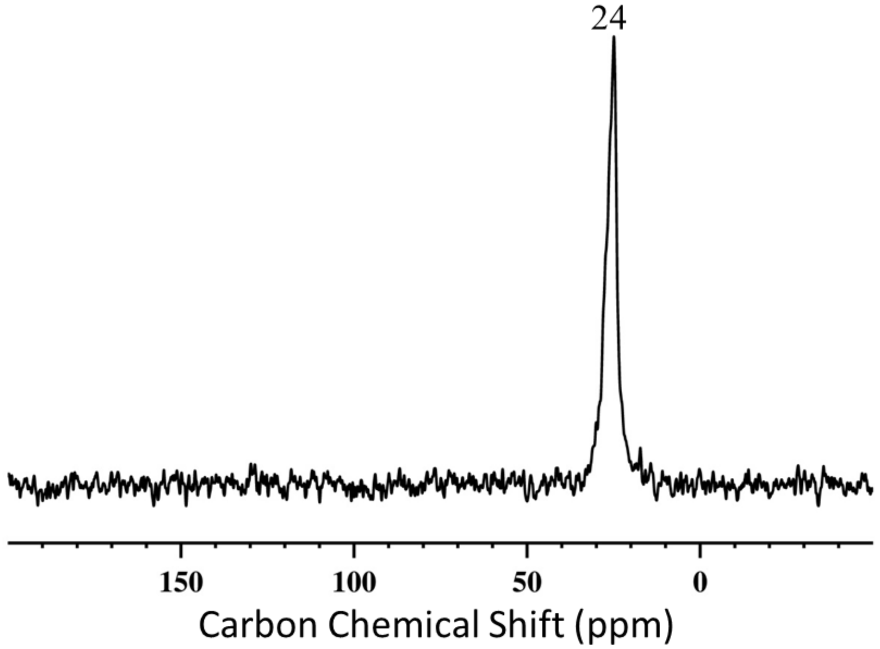

Figure S11: Solid-state ^{1}H-^{13}C CP-MAS NMR spectrum of 2.

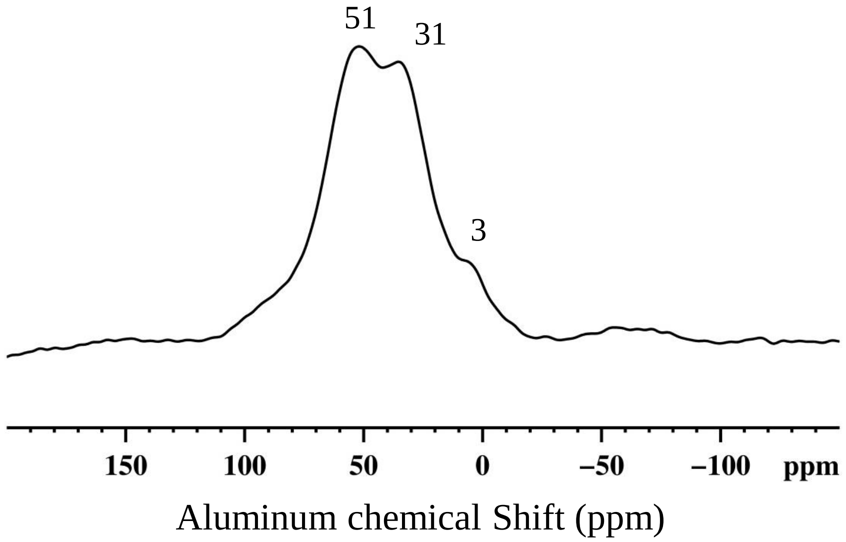

Figure S12: ^{27}Al projection of ^{1}H-^{27}Al J-HMQC MAS NMR spectrum of Figure 10b.

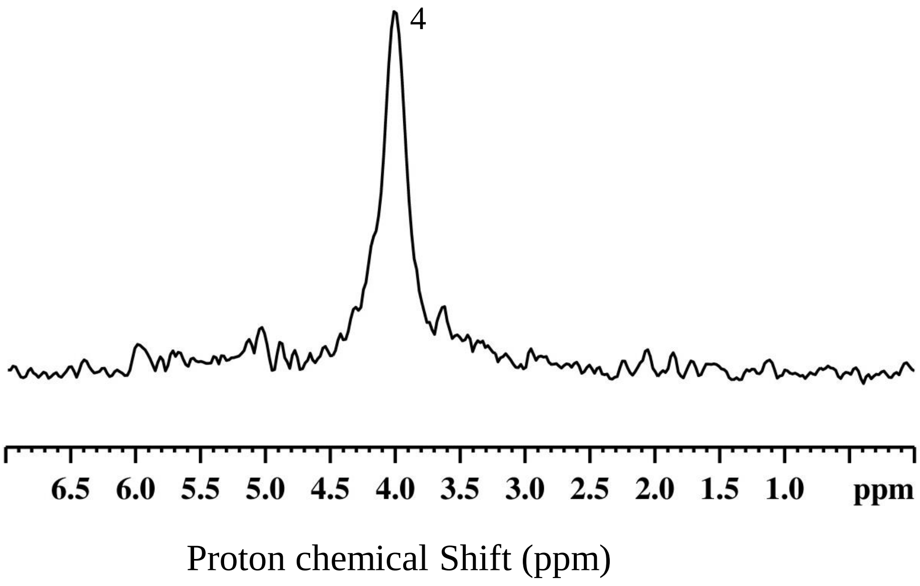

Figure S13: J-HMQC-filtered ^{1}H MAS NMR spectrum.

Computational details

MODEL AND Computational details:

Molecular structures and electronic energies for all the systems discussed here were calculated using the Gaussian 09 package. [4] All the DFT geometry optimizations were performed with the PBE functional [5,6] in connection with a split-valence basis set (SVP keyword in Gaussian) on all the atoms. No symmetry constraint was used in the geometry optimizations, and the final geometries were confirmed to be minimum or first order saddle point potential energy structures through frequency calculations. All systems have been treated with the spin-restricted formalism.

Transition states (TSs) calculations were approached through a linear transit [7] procedure using the forming Si–H, Al–H and Si–C bonds as the reaction coordinate of the β-H elimination, Al-H formation and alkyl transfer, respectively. For better energetics, single point energy calculations were performed using the hybrid PBE0 functional [8] and in connection with the triple-ζ TZVP basis set. [9,10] These PBE0 electronic energies were converted into the discusses free energies by addition of zero-point energy and thermal correction from the PBE/SVP frequency calculations.

We considered two models to mimic silica during the reaction with TIBA. The first model, used to investigate TIBA reactivity with a siloxane bridge, corresponds to a cluster model presenting only relaxed siloxane bridges, see (Figure S14a). The second model, used to investigate TIBA reactivity with a silanol, as well as the reactivity of monopodal {αSiO-Al-iBu$_2$} with a siloxane bridge, corresponds to a larger cluster model presenting a single silanol, in addition to relaxed siloxane bridges, see (Figure S14b). We are aware that the specific models we used can be only an approximation to the real situation occurring on the surface of silica after thermally treatment at 700 °C, which probably induces severe reconstruction of the surface. [11] Nevertheless, the amorphous nature of the silica makes impossible to derive more accurate models. On the other hand, there is ample precedent in the literature demonstrating the usefulness of silsesquioxane based cluster models to rationalize the reactivity of catalytically active species grafted on silica. [12-17]

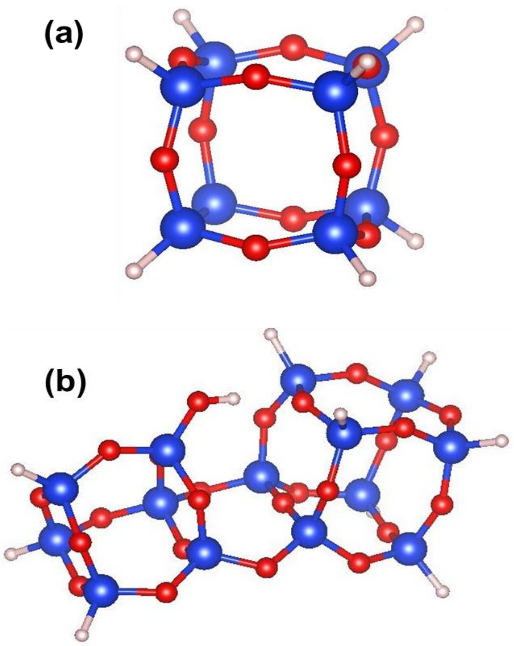

Figure S14: Representation of the two models considered. (a) is the model with only siloxane bridges and (b) is the model presenting silanol group with siloxane bridges.

Table S3: Energies of the isobutyl transfer from TIBA to Si of the model with Si-O-Si.

Isobutyl transfer	Entropy (PBE-svp)		Entropy (PBE1-tzvp)		Free energy (PBE1-tzvp)	
a0	-5689.9127	7.0	-5693.5653	2.8	-5693.0843	7.0
a1	-5689.9238	0.0	-5693.5697	0.0	-5693.0658	0.0
a1-a2	-5689.9039	12.5	-5693.5504	12.1	-5693.0400	16.2
a2	-5689.9438	-12.5	-5693.5910	-13.3	-5693.0890	-14.5

Table S4: Energies of the β-H transfer from one isobutyl group of TIBA to Si in the model with Si-O-Si with release of isobutene.

β-H transfer	Entropy (PBE-svp)		Entropy (PBE1-tzvp)		Free energy (PBE1-tzvp)	
a1	-5689.9238	0.0	-5693.5697	0.0	-5693.0658	0.0
a1-a2'	-5689.8694	34.2	-5693.5035	41.5	-5692.9986	42.2
a2'	-5689.8977	16.4	-5693.5437	16.4	-5693.0537	7.6
a3'	-5689.8940	18.7	-5693.5418	17.5	-5693.0679	-1.3

Table S5: Energies of the isobutyl transfer from TIBA to Si in the model with Si-OH.

Isobutyl transfer	Entropy (PBE/svp)		Entropy (PBE1-tzvp)		Free energy (PBE1-tzvp)	
b0	-7221.9105	12.3	-7226.6219	5.5	-7226.2088	59.9
b1	-7221.9301	0.0	-7226.6306	0.0	-7226.2254	4.5
b1-b2	-7221.9233	4.3	-7226.6165	8.9	-7226.2154	55.7
b2	-7221.9907	0.0	-7226.6940	0.0	-7226.3043	0.0
b2-b3	-7221.9596	19.5	-7226.6595	21.7	-7226.2650	24.6
b3	-7222.0187	-17.6	-7226.7218	-17.5	-7226.3312	-16.9

Table S6: Energies of the β-H transfer from one isobutyl group of TIBA to Si in the model with Si-OH with release of isobutene.

β-H transfer	Entropy (PBE/svp)		Entropy (PBE1-tzvp)		Free energy (PBE1-tzvp)	
b2	-7221.9907	-38.0	-7226.6940	0.0	-7226.3043	0.0
b2-b3'	-7221.9228	42.6	-7226.6176	48.0	-7226.2298	46.7
b3'	-7221.9747	10.0	-7226.6765	11.0	-7226.2940	6.4
b4'=b5"	-7221.9750	9.9	-7226.6783	9.9	-7226.3141	-6.2

Table S7: Energies of the β-H transfer from one isobutyl group of TIBA to Al forming Al-H in the model with Si-O-Si with release of isobutene, then the transfer of the hydride to Si

β-H transfer via Al-H	Entropy (PBE/svp)		Entropy (PBE1-tzvp)		Free energy (PBE1-tzvp)	
b2	-7221.9907	0.0	-7226.6940	0.0	-7226.3043	0.0
b2-b3"	-7221.9445	29.0	-7226.6447	30.9	-7226.2635	25.6
b3"	-7221.9583	20.3	-7226.6598	21.5	-7226.2778	16.6
b4"	-7221.9517	24.5	-7226.6566	23.5	-7226.2930	7.0
b4"-b5"	-7221.9350	35.0	-7226.6385	34.8	-7226.2765	17.4
b5"	-7221.9750	9.9	-7226.6783	9.9	-7226.3141	-6.2

Optimized geometries:

1. *Model A*

TIBA-INT0_1 **(a0_1)** **E=**-714.7354174 A.U.

C	2.283789	0.006470	-1.259837
H	2.364611	-0.865193	-1.949196
H	2.421645	0.908427	-1.900524
C	3.430042	-0.059669	-0.220770
H	4.396422	-0.169304	-0.767959
C	3.289582	-1.287779	0.690533
H	4.141773	-1.381663	1.395431
H	2.363807	-1.224372	1.309955
H	3.227815	-2.228348	0.105208
C	3.530119	1.228615	0.607740
H	4.385771	1.193749	1.313879
H	3.662762	2.119232	-0.040422
H	2.609863	1.395041	1.210152
Al	0.419524	0.012046	-0.535435
C	-0.640494	-1.654998	-0.788992
H	0.081655	-2.505530	-0.730006
H	-0.962528	-1.657189	-1.859731
C	-1.853081	-1.952581	0.118071
H	-2.589342	-1.124153	-0.002720
C	-2.565475	-3.254921	-0.282051
H	-1.882858	-4.125678	-0.175145
H	-3.458411	-3.449537	0.349159
H	-2.899248	-3.224123	-1.339714
C	-1.444433	-1.990412	1.598498
H	-0.693422	-2.789722	1.781032
H	-0.994058	-1.027525	1.924390
H	-2.310155	-2.190422	2.263458
C	-0.309359	1.660512	0.318893
H	-0.037178	1.607492	1.401584
H	0.283457	2.525263	-0.065296
C	-1.815863	1.971393	0.182842
H	-2.385125	1.125286	0.632769
C	-2.211611	3.245084	0.947157
H	-3.301824	3.446455	0.880985
H	-1.946454	3.168978	2.021809
H	-1.680793	4.131436	0.537029
C	-2.232215	2.070276	-1.292364
H	-3.314792	2.286637	-1.405911
H	-1.674620	2.882325	-1.807698
H	-2.028588	1.123886	-1.840182

SiOSi-INT0_2 **(a0_2)** E=-4975.177316 A.U.

Si	-2.179771	-0.543918	1.813741
Si	2.117791	0.504750	2.250608
Si	1.585890	-2.346019	0.859617
Si	-1.870663	2.421383	0.709700
Si	3.977999	-2.542088	-1.194684
Si	-5.266879	-1.239173	1.737051
Si	1.183528	2.431830	-0.105430
Si	-0.976423	-1.769634	-0.875515
Si	5.097646	1.022483	1.460638
Si	-3.677345	1.741647	-1.876061
Si	4.981390	0.443658	-1.670663
Si	0.575604	0.470253	-2.445980
Si	-5.341233	-0.901749	-1.414378
O	1.434502	-0.867248	1.603599
O	-1.667149	1.017040	1.581168
H	5.330852	-3.099280	-0.871240
O	-3.800132	-0.561442	2.163839
O	1.670392	1.839748	1.368142
O	-1.945025	-1.455655	0.436461
O	3.774347	0.358727	2.237332
O	0.477823	-2.422235	-0.381420
H	6.252392	0.098144	1.701905
H	-4.910421	2.549098	-2.151178
O	3.107221	-2.549560	0.233185
O	-3.081504	2.257790	-0.405885
H	-6.286685	-0.712722	2.691930
O	-0.442429	2.785895	-0.065371
O	4.124741	-0.985894	-1.776475
O	-5.730426	-0.769401	0.203611
H	1.937004	3.674750	-0.401376
O	-0.683716	-0.360020	-1.713448
H	5.355046	2.399431	1.987852
H	-2.642819	1.910803	-2.941566
H	3.262703	-3.342127	-2.237287
H	-5.157965	-2.736436	1.786303
O	1.469213	1.295967	-1.293215
H	-1.667054	-2.755361	-1.741505
O	4.798825	1.167708	-0.176519
O	-4.080962	0.120513	-1.811091
H	6.439271	0.147649	-1.886667
H	-4.883630	-2.293346	-1.729460
H	-0.048471	1.436954	-3.401780
H	4.435785	1.377316	-2.698418
H	1.511073	-0.480706	-3.119993
H	-6.565738	-0.516322	-2.186494
H	1.330306	-3.423457	1.849796
H	1.645733	0.668567	3.647079
H	-1.417874	-1.110439	2.954408
H	-2.230740	3.524082	1.635368

TIBA-SiOSi-R **(a1)** E=-5689.923821 A.U.

Si	-1.743795	0.823050	-1.678648
Si	2.062444	-1.957509	1.454634
Si	2.640485	-1.944495	-1.714602
Si	-1.567648	0.590844	1.354670

Si	5.668497	-1.144649	-1.779080
Si	-1.441854	4.022088	-1.653472
Si	1.462955	1.180790	1.677859
Si	1.295945	0.781193	-2.797360
Si	4.890999	-1.717364	2.778586
Si	-3.453517	3.123251	1.910342
Si	6.272225	0.438914	0.905260
Si	2.794957	2.685588	-0.823430
Si	-0.899358	5.001011	1.288605
O	1.964064	-1.998662	-0.200304
O	-2.057710	0.021308	-0.191658
H	6.836843	-2.064356	-1.594855
O	-1.945683	2.441363	-1.392587
O	1.706103	-0.424465	2.007263
O	-0.209842	0.466766	-2.164781
O	3.591374	-2.369497	1.952237
O	2.299133	-0.483066	-2.434080
H	6.009865	-2.707377	2.667700
H	-4.332436	3.211115	0.702338
O	4.292710	-2.080213	-1.616626
O	-2.707717	1.621098	1.943212
H	-2.681644	4.830861	-1.880815
O	-0.117905	1.387373	1.147968
O	5.698327	0.055886	-0.616297
O	-0.628887	4.574657	-0.308708
H	1.677851	1.956021	2.925703
O	1.881907	2.187033	-2.137007
H	4.508500	-1.443774	4.199096
H	-4.212207	3.259685	3.189017
H	5.675701	-0.470462	-3.115174
H	-0.501489	4.074134	-2.814020
O	2.493745	1.690851	0.490135
H	1.204498	0.950630	-4.269109
O	5.339515	-0.256990	2.102442
O	-2.321551	4.343685	1.854321
H	7.685381	-0.057405	1.032101
H	0.258355	4.494031	2.090756
H	2.392002	4.092377	-0.517595
H	6.170653	1.919730	1.064902
H	4.248387	2.558327	-1.139793
H	-1.028458	6.489386	1.380904
H	2.100071	-3.066713	-2.520244
H	1.073834	-2.910025	2.015691
H	-2.726084	0.325637	-2.664065
H	-1.418371	-0.573667	2.249086
C	-1.801717	-2.700479	-1.619301
H	-1.932885	-2.207753	-2.612251
H	-0.797020	-2.370716	-1.266966
C	-1.770262	-4.231259	-1.838301
H	-1.098531	-4.449149	-2.703602
C	-3.155214	-4.785985	-2.203805
H	-3.120477	-5.871400	-2.435543
H	-3.873916	-4.653458	-1.363466
H	-3.582431	-4.265098	-3.085781
C	-1.174055	-4.970540	-0.631336
H	-1.096711	-6.062315	-0.818414
H	-0.156429	-4.594032	-0.396444
H	-1.795678	-4.832011	0.278724
Al	-3.190394	-1.868279	-0.411634
C	-4.824143	-1.176527	-1.366200
H	-5.597674	-1.978269	-1.269762
H	-4.582452	-1.176254	-2.455274
C	-5.488950	0.171696	-1.005617
H	-4.723425	0.980285	-1.100546
C	-6.627251	0.526366	-1.977764
H	-7.436968	-0.233226	-1.924222
H	-7.079158	1.515453	-1.748654
H	-6.269234	0.552130	-3.027928
C	-5.995147	0.208258	0.444094
H	-6.761095	-0.578059	0.615407
H	-5.178111	0.032139	1.173065
H	-6.462530	1.185877	0.687584
C	-3.293322	-2.468861	1.511530
H	-2.244681	-2.660230	1.840329
H	-3.649926	-1.640060	2.170263
C	-4.132057	-3.727038	1.852836
H	-3.807095	-4.558715	1.183690
C	-3.879495	-4.194563	3.296378
H	-4.452207	-5.114773	3.540913
H	-2.804636	-4.409040	3.470190
H	-4.182737	-3.408052	4.021406
C	-5.634752	-3.516180	1.617768
H	-6.217140	-4.426840	1.869836
H	-6.020025	-2.687157	2.250108
H	-5.858126	-3.254146	0.563919

TIBA-SiOSi-TS-Alkyl **(a1-a2)** **E**=-5689.903908 A.U.

Si	-1.517600	-0.195817	-1.945154
Si	2.838933	-0.593817	2.549056
Si	2.905706	-2.734785	0.124683
Si	-1.333113	0.058496	1.147298
Si	5.737410	-2.481159	-1.201729
Si	-1.247931	3.016261	-2.659711
Si	1.420147	1.609552	0.818492
Si	1.447842	-1.372840	-2.497555
Si	5.624392	0.868071	2.394178
Si	-2.096607	2.718082	2.466183
Si	6.908350	0.354109	-0.442487
Si	2.828603	1.348668	-2.089283
Si	-1.463217	4.694928	0.022537
O	2.532196	-1.768123	1.417034
O	-2.131782	-0.332305	-0.342144
H	6.958637	-3.239658	-0.779820
O	-1.332356	1.374637	-2.372084
O	2.043870	0.811747	2.141097

O	-0.092765	-1.021248	-1.966395
O	4.471299	-0.310864	2.662324
O	2.240074	-2.143153	-1.271983
H	6.857822	0.437701	3.127606
H	-3.234887	3.039290	3.377577
O	4.555568	-2.804675	-0.063989
O	-2.274117	1.098744	2.055968
H	-2.580643	3.483185	-3.161017
O	-0.157590	1.150463	0.583561
O	6.080433	-0.846706	-1.257369
O	-0.832568	3.835733	-1.269365
H	1.475952	3.071934	1.071252
O	2.244638	0.018877	-2.929223
H	5.113228	2.198170	2.851752
H	-0.766186	2.936798	3.130420
H	5.259963	-2.871765	-2.565420
H	-0.160702	3.232035	-3.661976
O	2.334425	1.202566	-0.503722
H	1.374511	-2.256678	-3.687015
O	5.954333	1.006783	0.762816
O	-2.217282	3.693310	1.117515
H	8.156606	-0.225451	0.165315
H	-0.307969	5.388975	0.676185
H	2.254721	2.589798	-2.700584
H	7.203903	1.439073	-1.424967
H	4.320192	1.338970	-2.148788
H	-2.493795	5.655353	-0.486963
H	2.368664	-4.092997	0.389610
H	2.332983	-1.059151	3.864109
H	-2.532765	-0.851858	-2.801867
H	-0.569361	-1.061187	1.741361
C	-3.656465	-3.172155	-0.554530
H	-2.661924	-3.227141	-1.060927
H	-3.548261	-3.869209	0.309593
C	-4.734083	-3.747867	-1.501800
H	-4.383950	-4.748441	-1.852446
C	-4.937529	-2.894310	-2.761167
H	-5.670239	-3.357790	-3.454909
H	-5.315220	-1.880614	-2.507520
H	-3.987474	-2.766435	-3.321087
C	-6.064083	-3.986553	-0.771233
H	-6.815858	-4.463584	-1.434580
H	-5.926135	-4.647740	0.109735
H	-6.502893	-3.033437	-0.405180
Al	-3.771554	-1.344134	0.291654
C	-5.356358	-0.147402	0.010689
H	-5.593077	0.412290	0.944223
H	-6.202376	-0.865623	-0.099795
C	-5.401722	0.845852	-1.178064
H	-4.884123	0.391850	-2.058246
C	-6.843998	1.140277	-1.623470
H	-7.428201	1.581115	-0.787020
H	-6.879214	1.855585	-2.473024
H	-7.365654	0.213453	-1.940093
C	-4.672544	2.153375	-0.843304
H	-5.176257	2.678581	-0.003357
H	-3.626824	1.976034	-0.526507
H	-4.659615	2.849273	-1.708386
C	-3.100323	-1.678031	2.207931
H	-2.555483	-2.642675	2.081504
H	-2.521875	-1.117044	2.966085
C	-4.496198	-1.976812	2.848169
H	-5.250101	-2.200239	2.047288
C	-4.458139	-3.222326	3.748174
H	-5.450005	-3.429687	4.203709
H	-4.150330	-4.123875	3.179499
H	-3.728666	-3.081064	4.574324
C	-5.011675	-0.755935	3.623137
H	-6.048782	-0.908017	3.987223
H	-4.367266	-0.566949	4.508649
H	-4.990005	0.161111	3.002346

TIBA-SiOSi-P-Alkyl **(a2)** E=-5689.943765 A.U.

Si	-2.771158	-3.325943	0.212752
Si	2.718149	1.119610	1.691899
Si	2.164639	-2.033944	2.200397
Si	-0.538077	3.306026	-2.027096
Si	4.605484	-3.324016	0.678713
Si	-3.014106	-2.821175	-2.916099
Si	2.105231	1.554059	-1.405506
Si	0.378198	-3.295074	-0.171172
Si	5.727497	1.121276	0.771190
Si	-3.047932	1.899479	-0.667765
Si	5.552560	-1.169123	-1.427110
Si	1.281343	-1.295004	-2.440015
Si	-3.443938	0.265001	-3.456046
O	2.114404	-0.410809	1.863496
O	-2.922211	-1.952129	1.076054
H	5.994465	-3.487812	1.217687
O	-3.394579	-3.108976	-1.325951
O	2.203913	1.759381	0.244620
O	-1.171086	-3.801204	0.069581
O	4.380171	1.084024	1.756158
O	1.038973	-2.819408	1.277351
H	6.846486	0.460355	1.518134
H	-2.599749	0.836739	0.327231
O	3.691900	-2.618978	1.883777
O	-1.763916	2.914541	-0.941565
H	-4.203042	-3.220530	-3.737626
O	0.640717	2.124263	-1.926898
O	4.626329	-2.342984	-0.677220
O	-2.695981	-1.196529	-3.184824
H	3.214577	2.299275	-2.057603
O	0.373609	-2.009839	-1.233945

H	6.048963	2.539051	0.413833
H	-4.164125	2.696268	-0.082123
H	3.993803	-4.627996	0.270597
H	-1.778313	-3.567857	-3.314802
O	2.214302	-0.054039	-1.806693
H	1.172095	-4.428356	-0.722402
O	5.418317	0.309957	-0.657585
O	-3.573331	1.141929	-2.039173
H	6.993324	-1.597457	-1.385141
H	-2.596986	1.040585	-4.415597
H	0.332045	-0.746858	-3.455830
H	5.048276	-1.004657	-2.820388
H	2.237576	-2.292470	-3.017679
H	-4.838253	0.053907	-3.964724
H	1.864671	-2.267275	3.634141
H	2.215328	1.987025	2.785545
H	-3.502810	-4.459306	0.838755
H	-1.103793	3.230362	-3.413887
C	-1.349305	-0.054462	2.943218
H	-1.157901	-0.956025	3.574228
H	-0.558238	-0.100131	2.161264
C	-1.179653	1.222168	3.790179
H	-0.130935	1.241415	4.171828
C	-2.103069	1.233512	5.016167
H	-1.923378	2.124360	5.653276
H	-3.172541	1.254001	4.713316
H	-1.951445	0.332887	5.646569
C	-1.367147	2.489525	2.945115
H	-1.155249	3.410094	3.527783
H	-0.700263	2.491101	2.058242
H	-2.414891	2.571910	2.577061
Al	-3.043424	-0.480262	2.007158
C	-4.879195	0.143346	2.420028
H	-4.953364	1.244615	2.251050
H	-4.960273	0.038722	3.529253
C	-6.076739	-0.569062	1.751637
H	-5.978802	-1.661263	1.951713
C	-7.413931	-0.109369	2.354737
H	-7.571358	0.977122	2.179773
H	-8.274844	-0.648823	1.906200
H	-7.444473	-0.276166	3.451338
C	-6.077026	-0.387303	0.226987
H	-6.144329	0.688244	-0.046888
H	-5.158392	-0.803609	-0.234459
H	-6.942485	-0.899241	-0.242753
C	0.166774	5.012050	-1.686498
H	0.970851	5.117974	-2.452175
H	-0.605268	5.762025	-1.976120
C	0.726296	5.343696	-0.281988
H	1.335516	4.477391	0.064691
C	1.654565	6.566305	-0.339272
H	2.064534	6.807955	0.663200
H	2.512163	6.398857	-1.023158
H	1.106528	7.462667	-0.701505
C	-0.403260	5.559937	0.735301
H	0.002723	5.738243	1.751972
H	-1.014101	6.446766	0.459356
H	-1.082402	4.687235	0.784647

TIBA-SiOSi-TS-Beta-H **(a1-a2’)** E=-5689.869389 A.U.

Si	-1.745934	0.788108	-1.693953
Si	2.054666	-2.016210	1.494976
Si	2.701799	-2.450886	-1.630455
Si	-1.611139	0.786379	1.488738
Si	5.376099	-0.716824	-1.852823
Si	-1.832833	3.866607	-1.713437
Si	1.424141	1.202555	1.587289
Si	1.117321	0.073643	-2.707288
Si	4.858693	-1.340740	2.819858
Si	-3.398448	3.386500	2.033277
Si	5.851058	0.917494	0.824061
Si	2.471825	2.430402	-1.160265
Si	-0.964493	5.203173	1.046336
O	2.079601	-2.489734	-0.091956
O	-2.064979	-0.005266	0.047563
H	6.743355	-1.329726	-1.793977
O	-1.975528	2.334197	-1.035880
O	1.522715	-0.451963	1.627573
O	-0.159258	0.199275	-1.644487
O	3.577604	-2.164118	2.140497
O	1.792219	-1.439581	-2.584437
H	6.081279	-2.188005	2.633318
H	-4.348033	3.581373	0.886591
O	4.285479	-1.949852	-1.583231
O	-2.803690	1.827751	1.969583
H	-3.204080	4.356831	-2.079291
O	-0.172392	1.607461	1.294618
O	5.244452	0.448740	-0.660695
O	-1.184493	4.899197	-0.580199
H	1.858335	1.761101	2.893302
O	2.267373	1.203611	-2.280307
H	4.572393	-1.072822	4.264490
H	-4.086796	3.547542	3.350263
H	5.104026	-0.057070	-3.166589
H	-0.924764	3.848893	-2.905328
O	2.388587	1.837438	0.402558
H	0.715439	0.271851	-4.124420
O	5.063224	0.142010	2.077323
O	-2.186810	4.524809	1.960948
H	7.312998	0.568522	0.879794
H	0.352551	4.646201	1.489360
H	1.400342	3.464582	-1.340766
H	5.609013	2.381412	0.977879

H	3.833636	3.003289	-1.361392
H	-1.024550	6.686162	1.238255
H	2.642811	-3.816686	-2.204546
H	1.116509	-2.904405	2.224525
H	-1.778312	1.150243	-3.192683
H	-1.448894	-0.243253	2.543707
C	-1.848340	-2.733892	-1.375916
H	-2.142595	-2.343753	-2.379261
H	-0.800255	-2.382647	-1.246676
C	-1.856548	-4.279718	-1.421409
H	-1.285901	-4.597749	-2.326468
C	-3.272486	-4.855367	-1.567611
H	-3.259024	-5.959896	-1.678424
H	-3.895553	-4.624405	-0.675165
H	-3.792911	-4.438611	-2.456013
C	-1.130105	-4.884814	-0.211428
H	-1.077918	-5.991794	-0.277095
H	-0.093258	-4.498088	-0.133314
H	-1.649666	-4.631367	0.737328
Al	-2.915726	-1.747810	0.009915
C	-4.818618	-1.253886	-0.798764
H	-5.344603	-0.868079	0.093229
H	-5.123519	-2.275307	-1.093331
C	-4.651110	-0.327311	-1.875240
H	-3.089968	-0.020063	-1.946524
C	-4.702264	-0.833302	-3.298808
H	-5.770095	-0.894357	-3.605999
H	-4.189081	-0.152650	-4.007048
H	-4.270248	-1.848384	-3.390776
C	-5.081373	1.106929	-1.665680
H	-6.189757	1.146764	-1.750760
H	-4.807625	1.476864	-0.659079
H	-4.661824	1.794190	-2.426255
C	-3.167299	-2.304908	1.922655
H	-2.131585	-2.423343	2.321141
H	-3.581596	-1.433749	2.485837
C	-3.979958	-3.559646	2.320623
H	-3.589451	-4.431434	1.745648
C	-3.793083	-3.888427	3.811914
H	-4.340409	-4.810128	4.103152
H	-2.722627	-4.036749	4.063025
H	-4.170804	-3.058263	4.447399
C	-5.474846	-3.429726	1.992098
H	-6.043190	-4.318851	2.335845
H	-5.914877	-2.540947	2.494941
H	-5.659652	-3.321922	0.904532

TIBA-SiOSi-INT1-Beta-H **(a2')** E=-5689.897653 A.U.

Si	1.083641	1.203166	2.850764
Si	-2.314798	-2.118841	-1.334897
Si	-3.119432	-1.601528	1.751346
Si	2.389518	0.130019	-1.584482
Si	-6.270352	-1.509362	1.785358
Si	0.825743	4.096613	1.551892
Si	-0.671656	0.415403	-2.179381
Si	-2.101107	1.286674	2.524954
Si	-4.818842	-1.085571	-3.020545
Si	3.769825	3.048363	-1.374985
Si	-7.088315	-0.167641	-0.986519
Si	-2.706263	2.317581	-0.433122
Si	0.924209	4.522283	-1.590600
O	-2.470343	-1.569642	0.222289
O	1.982153	-0.033589	2.294449
H	-7.169507	-2.696754	1.617809
O	1.352804	2.557867	1.903933
O	-1.176672	-1.169795	-2.096086
O	-0.544353	0.811234	2.803955
O	-3.770952	-2.027203	-2.124349
O	-3.063203	-0.064415	2.376369
H	-5.672426	-2.024939	-3.816832
H	4.091484	3.192921	0.079858
O	-4.705373	-2.085184	1.678317
O	3.365402	1.455780	-1.699123
H	1.968697	5.041463	1.782832
O	0.791716	0.550944	-1.380786
O	-6.584679	-0.391636	0.586324
O	0.367706	4.190851	-0.054318
H	-0.498901	0.773319	-3.612261
O	-2.160551	2.201646	1.137467
H	-4.041959	-0.144171	-3.888211
H	4.930344	3.384289	-2.254347
H	-6.472895	-0.819180	3.097193
H	-0.365306	4.457910	2.385265
O	-1.773273	1.412080	-1.478857
H	-2.633283	2.094675	3.652738
O	-5.783784	-0.156657	-2.028457
O	2.519486	4.067419	-1.778720
H	-8.028985	-1.275003	-1.376649
H	0.069969	3.755240	-2.551246
H	-2.614310	3.750820	-0.842279
H	-7.731735	1.177968	-1.066287
H	-4.103327	1.787282	-0.518532
H	0.860933	5.996505	-1.847148
H	-2.367196	-2.561831	2.599362
H	-1.854669	-3.529488	-1.372524
H	1.434062	1.521363	4.265416
H	2.566147	-0.714142	-2.803018
C	1.088688	-2.770468	1.084668
H	0.647092	-3.048566	2.072390
H	0.299424	-2.150135	0.602515
C	1.343057	-4.047233	0.256376
H	0.368518	-4.578197	0.136264
C	2.294440	-5.020049	0.967846

H	2.403225	-5.969182	0.402725
H	3.310434	-4.582601	1.077877
H	1.929074	-5.275060	1.984314
C	1.844996	-3.719639	-1.157193
H	1.955950	-4.635107	-1.775099
H	1.144045	-3.041993	-1.688404
H	2.840820	-3.224146	-1.122901
Al	2.550526	-1.511257	1.549584
C	4.472521	-1.909923	1.814236
H	4.892237	-2.297617	0.854885
H	4.496059	-2.788961	2.502632
C	5.383772	-0.793010	2.369040
H	4.940293	-0.434861	3.326618
C	6.793909	-1.317596	2.684660
H	7.295149	-1.681185	1.760897
H	7.438806	-0.528368	3.125965
H	6.762193	-2.166417	3.398639
C	5.447106	0.413574	1.420999
H	5.826230	0.113429	0.419819
H	4.446188	0.869954	1.280859
H	6.120519	1.204282	1.812437
C	5.607360	-1.665562	-2.741908
H	4.776486	-2.337270	-3.012197
H	5.523585	-0.610932	-3.050602
C	6.688829	-2.121206	-2.072553
H	2.810926	-0.650259	-0.352996
C	6.816731	-3.560507	-1.641963
H	6.921550	-3.635119	-0.537089
H	5.942213	-4.167507	-1.946621
H	7.730823	-4.025987	-2.071160
C	7.845782	-1.223553	-1.714130
H	8.014259	-1.209970	-0.615060
H	8.790880	-1.595543	-2.166936
H	7.686823	-0.181371	-2.052186

TIBA-SiOSi-P-Beta-H **(a3’)** E=-5533.013245 A.U.

C	-1.207136	3.077903	0.188147
Al	-2.983898	2.264925	0.535375
C	-4.763970	3.108111	0.329238
C	-6.020887	2.326687	0.770507
C	-6.213117	1.045902	-0.054134
C	-1.022966	4.195897	-0.858876
C	-1.393274	3.720219	-2.271714
C	-1.792853	5.470544	-0.485741
O	-2.927074	0.870026	1.597008
Si	-2.185230	-0.225075	2.549914
O	-0.534140	0.068051	2.595142
Si	0.973448	-0.592535	2.751330
O	1.150086	-1.832032	1.657838
Si	1.857513	-2.397037	0.258423
O	1.066251	-1.843735	-1.103167
Si	0.365654	-0.841169	-2.200379
O	-1.150274	-0.409368	-1.640515
Si	-2.673044	0.020083	-2.143498
O	-3.748539	-1.223318	-2.036931
Si	-4.464657	-2.583496	-1.370848
O	-3.393127	-3.854522	-1.345347
Si	-1.906391	-4.474963	-0.904482
O	-1.413957	-3.882343	0.576714
Si	-2.041848	-3.371646	2.041929
O	-2.455834	-1.762555	1.948241
O	1.262525	0.539780	-2.434997
Si	2.339042	1.582808	-1.713276
O	3.885283	1.239869	-2.201433
Si	4.964489	0.085947	-2.741838
O	5.572484	-0.804879	-1.470392
Si	6.644748	-0.804285	-0.190886
O	5.909296	-0.239520	1.195168
Si	5.625773	1.113435	2.131760
O	4.274263	1.939909	1.598047
Si	2.623600	1.803911	1.484306
O	2.115655	0.590240	2.493720
O	2.194205	1.394908	-0.070297
C	-7.280780	3.205482	0.714795
H	6.778558	2.063116	2.008234
H	6.079011	0.821790	-3.421119
H	-4.918431	-2.272759	0.020517
H	-3.289634	-4.153384	2.331333
H	0.242966	-1.533531	-3.510015
H	4.267056	-0.872100	-3.656859
H	-5.592547	-2.962935	-2.274260
H	5.416962	0.642618	3.536221
H	-0.992319	-3.596513	3.086046
H	1.192879	-1.131926	4.118593
H	7.821456	0.070757	-0.526604
H	-0.875044	-4.074088	-1.911836
H	1.755654	-3.886103	0.263780
H	7.041925	-2.222786	0.055604
H	3.275886	-1.920301	0.193302
H	-2.065112	-5.962354	-0.829888
H	2.019656	3.108382	1.858830
H	2.022638	2.972080	-2.129933
H	-2.707816	-0.154466	3.945693
H	-2.661390	0.530867	-3.548394
H	-0.867804	3.441136	1.188255
H	-0.525874	2.225745	-0.034697
H	0.059460	4.467653	-0.885048
H	-1.600581	6.289729	-1.209556
H	-2.889455	5.289569	-0.473849
H	-1.504531	5.837892	0.521043
H	-1.184806	4.498586	-3.034911
H	-0.820885	2.812426	-2.556619
H	-2.476804	3.476116	-2.342093
H	-4.883301	3.446733	-0.728159

H	-4.694256	4.059102	0.911239
H	-5.874475	2.015284	1.830220
H	-7.482830	3.538998	-0.326205
H	-8.179839	2.660281	1.072269
H	-7.167623	4.116390	1.338245
H	-6.326195	1.281841	-1.135198
H	-5.348630	0.360083	0.056529
H	-7.121045	0.490363	0.260374
H	-3.142500	1.129422	-1.219063

2. *Model B*

TIBA+SiOH-INT0 **(b1)** E=-7380.030011 A.U.

Al	-1.314405	4.027948	-0.143982
C	-0.232154	3.602428	-1.781370
C	-3.142945	6.547270	-0.372801
C	-4.531028	7.070888	-0.776960
C	-2.723118	7.131972	0.983299
C	0.628284	4.699595	-2.447630
C	1.578535	5.375099	-1.446673
C	-0.235384	5.743298	-3.168999
C	-0.345292	4.024912	1.613703
C	-0.951140	3.327030	2.854189
C	0.039358	3.284436	4.030050
C	-2.275400	3.965029	3.299366
C	-3.077288	5.001619	-0.393656
H	3.729556	-3.645823	2.804800
H	-3.299683	2.687574	0.160435
H	-3.834918	-6.191241	0.068067
H	0.815872	-4.057711	-0.252307
H	0.431706	2.757323	-1.481101
H	-0.917818	3.164248	-2.543812
H	1.268678	4.223012	-3.229556
H	2.248457	6.107246	-1.944984
H	1.010453	5.928572	-0.665328
H	2.214805	4.632515	-0.922697
H	0.386971	6.505745	-3.682774
H	-4.862115	-3.109604	3.390532
H	-0.890828	5.270936	-3.929643
H	-0.896243	6.279454	-2.453305
H	-0.101375	5.081786	1.884639
H	0.647230	3.564228	1.402240
H	-1.168466	2.268189	2.580212
H	0.312339	4.312872	4.352203
H	-0.385665	2.757456	4.911290
H	0.972841	2.759405	3.743983
H	-2.130094	5.032667	3.571527
H	-3.040764	3.939980	2.496435
H	-6.709603	-0.545379	-0.281980
H	-2.700742	3.449679	4.186065
H	-3.477946	4.654220	-1.376974
H	-3.825005	4.638305	0.362835
H	-2.420007	6.924700	-1.134123
H	-4.562850	8.180975	-0.807170
H	-4.829852	6.696499	-1.777899
H	-5.303824	6.734592	-0.051519
H	-2.757603	8.241406	0.976697
H	-3.401406	6.782242	1.791510
H	-1.694906	6.826922	1.265880
H	-4.805682	-3.221375	-3.615185
H	3.291598	3.236598	2.083660
H	3.420420	2.772796	-2.748282
H	3.910895	-4.112813	-2.113058
H	6.971054	0.057935	2.535818
H	7.129074	-0.435974	-2.339883
O	5.030423	-1.555239	2.084075
O	3.822559	-3.201792	0.281395
O	-4.991273	-1.579553	1.324661
O	-4.778684	-1.818424	-1.434197
O	-4.365027	0.527417	-0.232285
O	-2.588418	-2.092912	-3.094599
O	-2.689904	-1.890569	2.877170
O	0.113505	-1.022731	-2.676793
O	-0.063502	-0.537729	2.519509
O	-2.068717	-0.388895	0.877457
O	-2.052202	0.094340	-1.679115
O	2.323772	-1.675972	1.942703
O	-2.436838	2.238031	0.008209
O	4.921086	0.826704	-2.019149
O	6.297537	-0.230212	0.077308
O	-3.639885	-4.211956	-1.550765
O	-3.689090	-3.881768	1.209843
O	-1.557788	-4.976285	0.010289
O	-1.217266	-2.508989	-0.969021
O	-0.663425	-2.845943	1.508698
O	5.091207	-1.868103	-1.727822
O	2.378927	-2.094403	-1.728562
O	0.901171	-0.549266	-0.068498
O	2.220200	0.580216	-2.163889
O	2.035243	1.012937	1.845790
O	4.749447	1.139594	1.844006
O	3.340704	2.144202	-0.248752
Si	1.411948	-0.757944	-1.652793
Si	-1.372589	-1.414359	1.974903
Si	-1.419038	-1.377548	-2.147923
Si	-2.710231	0.592130	-0.257402
Si	3.476335	1.624515	-1.820150
Si	5.895404	-0.425413	-1.523067
Si	-3.226079	-4.848109	-0.064668
Si	-0.568628	-3.639654	0.054993
Si	3.729690	-2.550439	1.810515
Si	3.812475	-2.852169	-1.344543
Si	1.307355	-0.436832	1.559060
Si	5.795072	-0.138921	1.659614
Si	3.357596	1.929813	1.397903
Si	-4.069817	-2.630345	2.236482
Si	-3.962956	-2.851309	-2.456610

Si	-5.273816	-0.881742	-0.157878

TIBA+SiOH-TS **(b1-b2)** E=-7380.023176 A.U.

C	0.262696	4.189975	-1.894195
C	1.056027	5.497666	-2.114823
C	0.144651	6.733007	-2.097349
Al	-0.869233	3.903208	-0.290090
O	-1.639911	2.179706	-0.480812
Si	-2.306519	0.688824	-0.646233
O	-1.708669	-0.129521	-1.956268
Si	-1.276331	-1.720338	-2.160635
O	-1.208227	-2.713428	-0.847914
Si	-0.825257	-3.710955	0.411930
O	-0.860581	-2.689953	1.722856
Si	-1.457509	-1.149537	1.916535
O	-2.032366	-0.351808	0.599339
C	-0.529749	4.458234	1.583305
C	-0.749173	3.460483	2.747045
C	-2.230618	3.115366	2.953836
C	-3.066094	4.623277	-0.934532
C	-4.125605	5.048804	0.091499
C	-5.216796	3.983872	0.262532
C	-4.744072	6.406706	-0.284347
C	2.211048	5.653963	-1.115178
C	-0.137364	3.988227	4.055238
O	-2.835916	-1.306251	2.839209
Si	-4.286597	-1.882687	2.177466
O	-4.039967	-3.318361	1.373575
Si	-3.646326	-4.527864	0.301845
O	-2.019109	-4.866607	0.511919
O	-0.107620	-0.280078	2.372745
Si	1.287979	-0.309719	1.453968
O	1.916191	1.220843	1.459127
Si	3.278208	2.051607	0.967357
O	3.361950	2.025898	-0.693316
Si	3.561383	1.212084	-2.130343
O	2.331396	0.105556	-2.315998
Si	1.545993	-1.185067	-1.654421
O	0.983222	-0.765080	-0.130419
O	0.305949	-1.632127	-2.676903
O	2.558313	-2.481040	-1.505583
Si	3.968315	-3.113277	-0.888891
O	5.252786	-2.168817	-1.352046
Si	6.009648	-0.689103	-1.387495
O	6.344181	-0.192703	0.163319
Si	5.756852	0.119803	1.686608
O	4.632558	1.342332	1.610945
O	-2.496246	-2.417958	-3.055651
Si	-3.995017	-2.846820	-2.396485
O	-3.902853	-4.085383	-1.284797
O	-3.952729	0.884046	-0.765603
Si	-5.058636	-0.349076	-0.522026
O	-4.682830	-1.561992	-1.594492
O	5.025143	0.426520	-2.131144
O	3.866877	-3.163298	0.770180
Si	3.744187	-2.272755	2.169615
O	2.347492	-1.373915	2.131366
O	5.048370	-1.250763	2.309157
O	-4.974458	-0.846161	1.067644
H	3.713991	-3.189874	3.329735
H	-2.500225	3.132690	-0.684968
H	-4.438607	-5.744649	0.594763
H	0.493761	-4.374897	0.290948
H	0.963926	3.322810	-1.919056
H	-0.420538	4.017299	-2.758100
H	1.516676	5.450915	-3.130133
H	2.820584	6.554556	-1.337704
H	1.833844	5.760812	-0.074838
H	2.885433	4.773589	-1.135229
H	0.704745	7.660640	-2.337738
H	-5.215563	-2.065111	3.315764
H	-0.683608	6.641249	-2.830335
H	-0.310795	6.879669	-1.091846
H	-1.107623	5.392541	1.785533
H	0.534986	4.790318	1.592988
H	-0.220186	2.514349	2.497355
H	-0.619258	4.942399	4.360561
H	-0.265468	3.265135	4.888315
H	0.949238	4.185139	3.944233
H	-2.814955	4.017095	3.239599
H	-2.691138	2.698388	2.035884
H	-6.421777	0.162885	-0.792292
H	-2.361453	2.365068	3.761233
H	-2.374947	5.474375	-1.157193
H	-3.514686	4.375268	-1.921957
H	-3.621426	5.174614	1.076509
H	-5.493638	6.734185	0.466401
H	-3.972951	7.201430	-0.358839
H	-5.258833	6.348172	-1.267525
H	-5.966358	4.295388	1.018876
H	-5.755436	3.814775	-0.694759
H	-4.795698	3.008774	0.576816
H	-4.842217	-3.272595	-3.533387
H	3.172517	3.448990	1.439096
H	3.520031	2.174421	-3.252541
H	4.151014	-4.486470	-1.408187
H	6.873952	0.530680	2.565818
H	7.274195	-0.803140	-2.147432

TIBA-SiOH-R **(b2)** E=-7221.990712 A.U.

Si	1.045905	-1.094264	-1.678296
Si	3.403008	-3.336280	1.446317

Si	3.414996	-3.106735	-1.784681
Si	0.986391	-1.326857	1.560784
Si	5.487432	-0.946731	1.551429
O	4.637093	-2.322442	1.923006
O	1.945528	-2.640745	1.842696
O	4.619563	-1.990866	-2.068479
O	1.933682	-2.420851	-2.116829
O	0.613178	-1.257215	-0.069512
O	1.982462	0.255336	-1.871825
O	1.804513	0.063972	1.952045
O	4.466710	0.373145	1.571979
H	3.531858	-4.627852	2.155105
Si	3.012378	1.147428	1.658236
O	2.706361	1.924137	0.140608
O	3.477169	-3.582023	-0.194011
O	-0.263079	-0.942503	-2.689648
O	-0.347012	-1.481550	2.543022
O	-0.223788	2.215063	-0.139569
Si	3.127748	1.355068	-1.440985
H	3.052063	2.194841	2.693693
H	3.152733	2.513836	-2.348866
Si	5.548816	-0.734032	-1.511707
O	4.621485	0.651533	-1.412052
H	3.606205	-4.278289	-2.666925
O	6.121607	-1.066233	0.016552
H	6.579191	-0.762180	2.533134
H	6.687389	-0.513205	-2.430811
C	1.293186	4.430865	1.784151
H	2.294984	4.211076	2.218032
H	1.403349	5.471844	1.393764
C	0.235490	4.448517	2.912764
H	0.561876	5.206272	3.664317
C	0.137550	3.107696	3.654166
H	-0.603028	3.159630	4.479526
H	-0.179074	2.284729	2.979546
H	1.110617	2.819428	4.103790
C	-1.139673	4.903202	2.401151
H	-1.864792	5.012012	3.234578
H	-1.075904	5.882923	1.883050
H	-1.574853	4.174021	1.684856
Al	1.065467	3.416125	0.080168
C	1.457062	4.445230	-1.578647
H	2.532138	4.742176	-1.577720
H	1.319730	3.824662	-2.493628
C	0.587104	5.720631	-1.727685
H	0.676068	6.318980	-0.789764
C	1.084647	6.611250	-2.877818
H	0.481610	7.539371	-2.970926
H	2.143663	6.908688	-2.731772
H	1.020948	6.071116	-3.847174
C	-0.899318	5.378686	-1.916286
H	-1.518344	6.294830	-2.011279
H	-1.047926	4.777703	-2.839510
H	-1.311900	4.783705	-1.074815
Si	-1.587802	1.399881	0.109532
Si	-2.448099	-3.300355	-0.246198
Si	-1.853322	-0.978429	2.004573
Si	-1.835142	-0.983877	-2.122029
Si	-5.324163	-2.638172	-0.164673
O	-4.056087	-3.730679	-0.236349
O	-2.030777	-2.573193	-1.679341
O	-3.227911	-0.927409	2.944029
O	-1.567871	0.587301	1.562937
O	-1.889506	0.160476	-0.949909
O	-2.938498	2.379773	0.063440
O	-3.147033	-0.600806	-3.072731
O	-5.138671	-1.574748	-1.429138
H	-1.620023	-4.504517	0.001067
Si	-4.680045	-0.311920	-2.411941
O	-4.755205	1.063127	-1.477565
O	-2.238387	-2.105288	0.871498
Si	-4.500224	1.788242	0.004291
H	-5.607496	-0.177291	-3.558397
H	-5.452222	2.914414	0.147467
Si	-4.745984	-0.615145	2.257154
O	-4.701284	0.720355	1.268314
O	-5.295574	-1.877880	1.317750
H	-6.603468	-3.371988	-0.303866
H	-5.678629	-0.404812	3.387935

TIBA-SiOH-TS-Alkyl **(b2-b3)** E=-7221.959617 A.U.

C	2.114637	3.426109	1.625915
C	1.718207	3.388398	3.146673
C	2.645972	2.588894	4.074206
Al	1.371263	3.134257	-0.346350
O	-0.206019	2.407230	-0.234901
Si	-1.572663	1.550381	-0.328034
O	-1.592133	0.652968	-1.725824
Si	-2.010899	-0.907767	-2.066571
O	-3.368252	-0.819271	-3.029996
Si	-4.872358	-0.422848	-2.354099
O	-5.510219	-1.639183	-1.405150
Si	-5.515401	-2.277740	0.136952
O	-4.341998	-3.472237	0.252108
Si	-2.725279	-3.057984	0.288180
O	-2.291449	-2.299037	1.697097
Si	-1.916225	-0.716045	2.049897
O	-3.193633	-0.152547	2.964426
Si	-4.716489	0.122381	2.281927
O	-5.098995	-1.146740	1.280601
Si	2.973057	1.399793	1.080427
O	1.607169	0.608496	1.659591
Si	0.949611	-0.912643	1.607354
O	1.976725	-2.089270	2.149640
Si	3.295669	-3.053799	1.814405
O	3.220428	-3.643239	0.260018
Si	3.094453	-3.572665	-1.390462

O	4.285169	-2.602776	-2.034298
Si	5.298065	-1.295894	-1.869549
O	5.956280	-1.280171	-0.339079
Si	5.207934	-0.849013	1.098036
O	4.719891	-2.191694	1.966763
C	1.712806	4.733375	-1.433976
C	0.803851	5.967559	-1.215629
C	1.359255	7.205876	-1.937566
O	2.717777	1.821657	-0.653014
Si	2.960086	0.838277	-2.071634
O	1.712455	-0.232082	-2.086506
Si	0.799195	-1.518426	-1.581509
O	0.470683	-1.311227	0.043566
C	-0.646309	5.694678	-1.643066
O	3.836305	-0.042647	0.634285
O	4.442102	0.123633	-2.167189
O	1.613505	-2.933747	-1.809259
O	-0.567207	-1.607041	-2.534524
O	-2.531034	-1.859482	-0.833470
O	-4.751836	0.922705	-1.382845
Si	-4.484675	2.072500	-0.208662
O	-2.893206	2.565448	-0.307574
O	-4.768061	1.489053	1.330392
O	-0.362411	-0.837334	2.642174
O	-1.800947	0.416373	0.855444
C	0.251081	2.979403	3.345250
H	3.309048	-4.179509	2.775299
H	4.106259	1.982085	1.858938
H	2.819465	1.806737	-3.185019
H	3.200957	-4.932912	-1.963437
H	6.159597	-0.068189	1.929822
H	6.387224	-1.379808	-2.868964
H	3.036444	4.038290	1.498713
H	1.310323	4.159328	1.297860
H	1.805462	4.448164	3.479611
H	2.346026	2.765304	5.127407
H	2.577019	1.495701	3.901618
H	3.708229	2.891925	3.976384
H	-0.032451	3.064822	4.414088
H	-0.436350	3.629159	2.764569
H	0.080925	1.933756	3.026987
H	2.775711	5.016991	-1.249250
H	1.682853	4.450759	-2.513477
H	0.792825	6.206169	-0.124187
H	0.727937	8.101760	-1.760181
H	2.389711	7.445461	-1.602785
H	1.396865	7.034260	-3.034965
H	-1.292531	6.578878	-1.465491
H	-0.694433	5.461899	-2.728943
H	-1.092578	4.836001	-1.101482
H	-1.908500	-4.272120	0.049724
H	-5.785857	-0.174474	-3.493245
H	-5.369378	3.238996	-0.437976
H	-6.851607	-2.858867	0.407932
H	-5.678432	0.266543	3.399125

TIBA-SiOH-P-Alkyl **(b3)** E=-7222.018694 A.U.

Si	-0.473021	-2.253593	1.318708
Si	-2.953825	-2.215643	-2.548411
Si	-2.616445	-4.087740	-0.024873
Si	-0.917553	-0.118014	-1.124105
Si	-5.321397	-0.605190	-1.264896
O	-4.462036	-1.546821	-2.344306
O	-1.823405	-1.061121	-2.114196
O	-3.866625	-3.484825	0.878278
O	-1.152357	-3.563946	0.570581
O	-0.379246	-1.008772	0.157106
O	-1.386204	-1.761665	2.583599
O	-1.949692	1.172911	-0.584873
O	-4.614318	0.909059	-1.189874
H	-2.760964	-2.578475	-3.969548
Si	-3.238533	1.705331	-1.688779
O	-2.408970	0.720879	2.220296
O	-2.767469	-3.560610	-1.602486
Si	4.516768	1.744637	-1.899883
Si	5.229940	-1.023489	1.787012
O	4.567968	2.405723	-0.371714
O	4.920900	0.607959	1.718723
H	5.255444	2.631324	-2.828427
H	5.216512	3.153289	1.982899
Si	4.412295	2.131547	1.272546
O	2.809166	2.282804	1.729546
H	6.255220	-1.318417	2.813476
O	3.859158	-1.919194	2.202819
O	2.954994	1.580366	-2.559680
O	1.036584	-2.702957	1.862945
O	0.251815	0.657020	-2.016449
Si	1.911752	0.483907	-1.868371
Si	2.353396	-1.734067	1.517510
O	2.234554	0.678127	-0.272004
O	1.827756	-0.250935	2.062379
Si	1.674701	1.174560	1.222209
O	0.179190	1.770550	1.203572
Si	-2.598430	-0.682485	3.022042
H	-2.784867	1.197964	-3.016276
H	-2.562550	-0.534694	4.498469
Si	-4.849900	-2.284666	1.485581
O	-4.059898	-1.406074	2.635679
H	-2.643873	-5.567075	-0.015563
O	-5.314557	-1.288910	0.233963
H	-6.712452	-0.460558	-1.754126
H	-6.056244	-2.906160	2.080333
C	-3.531779	3.551181	-1.643870
H	-4.614741	3.656119	-1.879148
H	-3.421801	3.911198	-0.601583
C	-2.687589	4.419715	-2.615269
H	-2.924847	5.477701	-2.363150

C	-3.091048	4.205630	-4.081871
H	-2.539892	4.900428	-4.747710
H	-2.863948	3.174947	-4.426506
H	-4.175557	4.381867	-4.236566
C	-1.176142	4.230490	-2.414304
H	-0.601012	4.914451	-3.070858
H	-0.870904	4.437619	-1.368563
H	-0.848263	3.196206	-2.657081
Al	-1.577660	1.927333	1.260582
C	-2.140205	3.795948	1.539801
H	-1.779500	4.418792	0.691277
H	-3.250444	3.899360	1.548334
C	-1.573582	4.396657	2.854733
H	-0.472538	4.221231	2.862056
C	-1.793019	5.916576	2.915720
H	-1.363477	6.356000	3.840721
H	-1.325496	6.430162	2.050208
H	-2.877904	6.159051	2.902407
C	-2.162793	3.716455	4.099809
H	-1.720893	4.124499	5.032079
H	-3.261637	3.876511	4.151559
H	-1.995390	2.619888	4.095549
O	5.809089	-1.454804	0.285342
O	5.235014	0.246173	-1.813230
H	7.017612	-1.605780	-1.972189
Si	5.704964	-1.284503	-1.365852
O	4.561980	-2.363381	-1.938163
O	2.606720	-2.079445	-0.080027
O	2.236157	-1.028366	-2.462936
Si	2.919007	-2.337563	-1.678585
H	2.328711	-3.599053	-2.183189

TIBA-SiOH-TS-Beta-H **(b2-b3’)** E=-7221.922797 A.U.

Al	1.380538	3.092496	-0.051742
C	1.941200	4.177390	1.728061
C	3.269152	4.023442	2.169680
C	3.547300	3.543767	3.561737
C	4.389003	4.702393	1.432574
C	0.936910	5.741555	-1.437200
C	1.218000	6.614485	-2.671760
C	-0.568307	5.693305	-1.133772
C	1.546756	4.330089	-1.591068
H	2.816357	-5.060204	1.994075
H	1.131666	3.937597	2.439596
H	5.340828	4.145457	1.520686
H	4.154744	4.866444	0.363252
H	4.549760	5.701975	1.897078
H	-5.383354	0.246289	3.849764
H	2.622026	4.415733	-1.872421
H	3.384227	4.405487	4.249003
H	2.836490	2.751309	3.870845
H	4.585072	3.185588	3.693942
H	3.901179	2.183116	1.551677
H	1.760088	5.075367	1.101114
H	1.063617	3.824533	-2.460616
H	1.435848	6.247925	-0.572730
H	0.825083	7.645029	-2.544424
H	0.736161	6.180483	-3.573749
H	2.305580	6.688037	-2.879086
H	-6.946244	-2.603312	0.549243
H	-2.127023	-4.129708	-0.322321
H	-0.799468	5.134020	-0.203193
H	-1.118662	5.185274	-1.954263
H	-0.994135	6.712205	-1.027467
H	-5.267097	3.556880	0.348036
H	-6.065114	0.462044	-3.065110
H	2.209045	1.185666	2.641718
H	2.842988	-4.792585	-2.779671
H	2.858118	1.932682	-2.753069
H	6.350727	-1.777899	2.281519
H	6.205508	-1.231643	-2.596983
O	2.800683	-3.984399	-0.337792
O	-4.584537	1.570438	1.805492
O	4.083911	-2.839142	1.799010
O	-4.887010	1.365958	-0.946445
O	-2.837183	2.772263	0.082069
O	-3.625200	-0.202949	-2.845927
O	-3.016357	-0.354082	3.153173
O	-0.875095	-1.256784	-2.773881
O	-0.214461	-0.884009	2.392136
O	-1.992273	0.416223	0.944795
O	1.355640	-2.973077	1.716365
O	-1.596905	0.940396	-1.542827
O	4.190259	0.046832	-1.632409
O	5.580642	-1.728052	-0.159648
O	-5.651277	-1.178551	-1.137690
O	-4.468039	-3.219322	0.237519
O	-5.206993	-1.013204	1.592743
O	1.343919	-2.781243	-2.332874
O	0.093294	-1.569594	-0.255517
O	4.061378	-2.585930	-2.281173
O	-2.239226	-2.332423	1.553728
O	-2.714031	-1.620279	-0.926855
O	1.480270	-0.095157	-2.095719
O	2.181495	-0.538199	0.967553
O	4.533710	-0.072983	1.747250
O	2.554858	1.652298	-0.213337
O	-0.181258	2.388255	0.307813
Si	0.517465	-1.426750	-1.864304
Si	2.765683	0.869879	-1.724089
Si	5.046579	-1.387182	-1.687110
Si	-1.590121	1.673951	-0.045319
Si	-2.198592	-0.528442	-2.044529
Si	-1.846576	-0.794406	2.045427
Si	5.136690	-1.593650	1.449488
Si	3.117776	0.835307	1.479940

Si	-4.557890	0.117749	2.625914
Si	-4.431109	2.332831	0.328078
Si	-5.072885	0.111029	-2.022529
Si	2.763801	-3.574470	-1.942412
Si	0.859210	-1.461535	1.239174
Si	2.779635	-3.746080	1.311652
Si	-2.834056	-2.901417	0.109683
Si	-5.612953	-1.999748	0.314954

TIBA-SiOH-INT1-Beta-H **(b3')** E=-7221.974712 A.U.

Si	0.698217	-1.133871	-1.981131
Si	2.963812	-3.713773	1.042533
Si	2.945656	-3.287585	-2.093118
Si	1.077224	-1.184582	1.254468
Si	5.457131	-1.718998	1.416433
O	4.383199	-2.987470	1.518474
O	1.676116	-2.721410	1.416700
O	4.145509	-2.146499	-2.229810
O	1.456595	-2.571655	-2.293331
O	0.540257	-0.972054	-0.309020
O	1.557480	0.125801	-2.586061
O	2.289960	-0.096520	1.609748
O	4.893129	-0.467719	2.367807
H	2.804293	-4.996047	1.763553
Si	3.367806	-0.050638	2.910868
O	2.190755	2.474515	-1.341033
O	3.009546	-4.005904	-0.591986
Si	-4.542510	-0.305190	2.629432
Si	-5.089429	-0.299990	-2.010621
O	-4.709154	1.144028	1.827316
O	-4.950583	0.947814	-0.920671
H	-5.350244	-0.270921	3.870594
H	-5.575875	3.075894	0.390357
Si	-4.614280	1.948249	0.366823
O	-3.070719	2.550714	0.168384
H	-6.126721	-0.004162	-3.025749
O	-3.643148	-0.528373	-2.861685
O	-2.952364	-0.633915	3.109761
O	-0.774922	-1.193220	-2.767972
O	-0.118981	-0.933590	2.395432
Si	-1.742169	-0.940980	2.005203
Si	-2.182874	-0.647007	-2.064268
O	-1.911298	0.302225	0.947661
O	-1.809001	0.888843	-1.572075
Si	-1.722369	1.595432	-0.068307
O	-0.365810	2.450936	0.126646
Si	2.741503	1.267454	-2.284144
H	2.910162	-1.004279	3.972325
H	3.207869	1.767741	-3.606451
Si	5.002246	-0.843762	-1.647991
O	4.016166	0.484111	-1.512701
H	3.128071	-4.329902	-3.127805
O	5.619465	-1.203618	-0.148989
H	6.782785	-2.147748	1.920438
H	6.140158	-0.582496	-2.564282
C	1.977650	3.479622	1.645823
H	1.848764	2.383042	1.661508
H	1.178872	4.090209	2.100794
C	3.201896	4.030814	1.368828
H	3.444416	1.354753	3.407135
C	4.364230	3.196612	0.920047
H	4.890413	3.675519	0.068940
H	5.102607	3.128720	1.749415
H	4.071936	2.174456	0.620603
C	3.449409	5.504361	1.508166
H	3.785750	5.935437	0.541602
H	2.558023	6.059143	1.855386
H	4.280524	5.677271	2.225443
Al	1.000368	3.402375	-0.448015
C	0.751538	5.260377	-1.054122
H	1.778853	5.663415	-1.215172
H	0.322043	5.194889	-2.083236
C	-0.082323	6.266357	-0.229500
H	0.295551	6.261344	0.821773
C	0.087009	7.700783	-0.756918
H	-0.487686	8.432765	-0.151025
H	1.151379	8.016158	-0.749600
H	-0.273912	7.777419	-1.805228
C	-1.567210	5.878163	-0.183676
H	-2.154168	6.594309	0.428077
H	-2.002041	5.883860	-1.206834
H	-1.727872	4.863740	0.232242
O	-5.557697	-1.639265	-1.131917
O	-5.101522	-1.483387	1.596051
H	-6.685625	-3.220465	0.538205
Si	-5.425286	-2.477787	0.302834
O	-4.161118	-3.572282	0.187082
O	-2.516907	-1.789164	-0.926581
O	-1.985076	-2.505596	1.492164
Si	-2.565973	-3.111390	0.058656
H	-1.757160	-4.264402	-0.403256

TIBA-SiOH-P-Beta-H **(b4'=b5")** E=-7065.094164 A.U.

Al	-1.389578	2.587135	1.064715
C	-1.839516	4.493868	0.919865
C	-1.222866	5.359924	-0.203220
C	0.298087	5.504491	-0.042724
O	-2.292567	1.695440	2.275754
Si	-2.890730	0.292359	2.839512
O	-4.211286	-0.164955	1.906448
Si	-5.245341	-1.325322	1.350326
O	-5.950601	-0.759153	-0.052968
Si	-5.593312	-0.119082	-1.535117
O	-4.673093	1.260578	-1.325442

Si	-3.318020	2.165043	-1.586039
O	-1.984452	1.584932	-0.602818
Si	-1.091966	0.181696	-1.094570
O	0.165286	0.757284	-2.011317
Si	1.777371	0.321288	-1.884664
O	1.826712	-1.281776	-2.304755
Si	2.351640	-2.577999	-1.384155
O	2.125005	-2.125805	0.185462
Si	1.965310	-1.550010	1.728243
O	1.652140	0.044417	2.095918
Si	1.713707	1.360213	1.086441
O	0.310533	2.139864	0.954948
O	-2.135610	-0.629295	-2.070498
Si	-3.197507	-1.868559	-2.453869
O	-3.072486	-3.079232	-1.334345
Si	-3.146221	-3.505637	0.278938
O	-1.712474	-3.077772	1.004741
Si	-0.905264	-1.746303	1.563078
O	0.537106	-2.276915	2.204019
O	-0.677992	-0.707559	0.229548
O	-1.769944	-0.956334	2.709514
O	-4.722835	-1.206679	-2.454792
O	-4.418142	-2.732108	1.009983
O	2.947686	1.165494	-2.708649
Si	4.534717	1.185712	-2.088888
O	5.045956	-0.379122	-1.845385
Si	5.276423	-1.904917	-1.225890
O	3.967881	-2.850989	-1.663497
O	4.709770	2.000690	-0.646588
Si	4.549611	1.941990	1.018118
O	2.988572	2.361175	1.461081
O	4.855270	0.424013	1.634640
Si	4.923807	-1.218283	1.882077
O	5.388884	-1.896527	0.433447
O	3.450918	-1.851907	2.414642
O	2.198191	0.596792	-0.321462
C	-1.893107	6.741098	-0.280303
H	-2.883800	-2.396349	-3.799688
H	5.362333	1.854518	-3.118876
H	5.496014	2.919045	1.604589
H	5.924030	-1.544775	2.923442
H	-2.849589	2.081190	-3.000912
H	-3.290988	0.374524	4.267051
H	-3.320365	-4.971191	0.379288
H	-6.849927	0.217187	-2.241829
H	-6.326966	-1.636252	2.315142
H	-3.569024	3.544708	-1.100524
H	-2.949636	4.585159	0.909507
H	-1.547852	4.929878	1.906465
H	-1.404817	4.854815	-1.183324
H	-1.465064	7.362326	-1.095103
H	-2.985612	6.655377	-0.456437
H	-1.753897	7.295134	0.672955
H	0.740367	6.087192	-0.877024
H	0.540483	6.038488	0.901471
H	0.806430	4.520600	-0.000600
H	6.508846	-2.496292	-1.795680
H	1.581386	-3.791345	-1.743536

TIBA-SiOH-TS1-AlH **(b2-b3”)** E=-7221.944556 A.U.

C	-0.650588	5.178752	-1.166645
C	0.321101	5.410263	-2.346064
C	1.668581	5.981131	-1.880998
Al	0.046714	4.021195	0.281204
O	0.902754	2.538138	-0.149535
Si	2.035175	1.441377	0.187411
O	1.756136	0.670772	1.640655
Si	1.716235	-0.938190	2.043417
O	1.870299	-2.100864	0.888298
Si	1.806557	-3.292722	-0.251665
O	1.565109	-2.473186	-1.676956
Si	1.754071	-0.869103	-2.074051
O	2.130901	0.161686	-0.853887
O	3.116101	-0.773232	-3.029454
Si	4.668212	-0.833395	-2.350251
O	4.830907	-2.178605	-1.382708
Si	4.757741	-3.288749	-0.147134
O	3.279618	-4.069059	-0.251998
O	5.046128	0.479902	-1.396688
Si	4.954880	1.197303	0.108194
O	3.563156	2.117087	0.212194
O	4.917555	0.081008	1.344445
Si	4.630280	-1.239892	2.311525
O	4.885866	-2.581672	1.356856
O	3.074452	-1.189132	2.977839
O	0.238223	-0.409973	-2.593987
Si	-1.110016	-0.566308	-1.624231
O	-2.027663	0.790805	-1.823880
Si	-3.427677	1.567841	-1.375883
O	-3.444861	1.769142	0.273262
Si	-3.463775	1.148431	1.812107
O	-4.795227	0.172190	2.002708
Si	-5.653366	-1.149599	1.470741
O	-6.098045	-0.931122	-0.115945
Si	-5.611458	-0.737985	-1.694447
O	-4.747937	0.675359	-1.845604
O	-1.953492	-1.903007	-2.103321
Si	-3.270259	-2.901941	-1.941647
O	-3.265609	-3.591757	-0.427671
Si	-3.321985	-3.324265	1.212023
O	-2.004044	-2.421752	1.674887
Si	-1.157556	-1.006850	1.585167
O	0.156491	-1.148096	2.603774
O	-0.706437	-0.744158	-0.006624
O	-2.082773	0.264296	2.098464
O	-4.666388	-2.025310	-2.153945

O	-4.719678	-2.518067	1.609877
C	-0.313941	6.307749	-3.420769
C	1.037860	4.983594	1.852175
C	-0.092007	4.471902	2.570358
C	-1.183251	5.436332	2.981815
C	0.078492	3.250476	3.445105
H	-3.202599	-3.970960	-2.962108
H	5.613292	-0.891365	-3.489115
H	6.132007	2.082877	0.274229
H	5.570182	-1.268620	3.455876
H	-3.478437	2.890582	-2.036936
H	-3.533193	2.254075	2.794183
H	-3.289142	-4.624153	1.918137
H	5.842256	-4.286354	-0.300699
H	0.728175	-4.283427	-0.022443
H	-6.805930	-0.672845	-2.565883
H	-6.869431	-1.303067	2.300497
H	-1.599008	4.732896	-1.539903
H	-0.940303	6.158237	-0.718493
H	0.524790	4.420662	-2.815796
H	0.355958	6.434277	-4.297181
H	-1.272779	5.885769	-3.785854
H	-0.528914	7.319473	-3.013426
H	2.352712	6.162270	-2.735474
H	1.530436	6.949620	-1.352651
H	2.190692	5.289652	-1.184653
H	2.009849	4.477440	1.982664
H	1.081491	6.068284	1.657891
H	-1.145839	3.669080	1.408762
H	-0.885185	2.750453	3.660086
H	0.767742	2.504946	3.004755
H	0.518827	3.590884	4.408526
H	-0.839972	5.958485	3.902270
H	-1.368012	6.209652	2.211235
H	-2.135602	4.919964	3.209678

TIBA-SiOH-INT1-AlH **(b3")** E=-7221.958275 A.U.

Si	-1.047770	-1.187860	1.681357
Si	-3.579627	-3.091781	-1.534976
Si	-3.513002	-3.159912	1.695574
Si	-1.157049	-1.146783	-1.574777
Si	-5.621489	-0.624339	-1.370556
O	-4.730582	-1.933748	-1.866413
O	-2.072793	-2.485228	-1.899675
O	-4.725289	-2.105136	2.136863
O	-2.031991	-2.471706	2.016709
O	-0.711767	-1.180742	0.039073
O	-1.815021	0.227605	2.080758
O	-2.063822	0.205758	-1.863468
O	-4.665477	0.749219	-1.370089
H	-3.830178	-4.291523	-2.362976
Si	-3.135646	1.371394	-1.415548
O	-2.669086	1.940914	0.143200
O	-3.641306	-3.497724	0.074545
Si	4.570883	-0.685090	-2.514406
Si	4.727277	-0.632715	2.158574
O	4.716919	0.761473	-1.702208
O	4.715603	0.618349	1.063692
H	5.478194	-0.680805	-3.684627
H	5.516953	2.696626	-0.233788
Si	4.524023	1.597164	-0.270899
O	2.986018	2.250270	-0.204418
H	5.690339	-0.369559	3.252279
O	3.211391	-0.815739	2.892669
O	3.015343	-0.972738	-3.121681
O	0.317325	-1.334960	2.621974
O	0.132337	-1.115540	-2.622409
Si	1.717427	-1.220812	-2.108764
Si	1.809792	-0.899392	1.995956
O	1.884542	-0.008997	-1.017167
O	1.538563	0.637177	1.444550
Si	1.601675	1.323011	-0.073002
O	0.269207	2.162272	-0.431411
Si	-3.000285	1.320432	1.724527
H	-3.075566	2.531730	-2.326089
H	-3.083394	2.418067	2.702786
Si	-5.531161	-0.718846	1.704462
O	-4.464954	0.557162	1.655661
H	-3.641816	-4.407031	2.480118
O	-6.179860	-0.873405	0.178051
O	5.207516	-1.990808	1.319703
O	5.004511	-1.877131	-1.436703
H	6.418217	-3.651140	-0.212968
Si	5.172645	-2.856712	-0.103986
O	3.861346	-3.898345	-0.073401
O	2.139643	-2.117573	0.940742
O	1.840518	-2.783391	-1.557514
Si	2.270268	-3.407951	-0.078898
Al	-0.954459	3.285777	0.205294
H	1.404747	-4.555007	0.284174
H	-6.767741	-0.424705	-2.285028
H	-6.612957	-0.460947	2.681208
C	-1.548419	4.752572	-0.986244
H	-2.664041	4.751895	-0.974246
H	-1.274982	5.702071	-0.466735
C	-1.043630	4.822677	-2.447103
H	-1.257627	3.845842	-2.941418
C	-1.785332	5.906470	-3.246586
H	-1.446131	5.943328	-4.303433
H	-2.881814	5.733485	-3.248626
H	-1.609625	6.910420	-2.802932
C	0.474712	5.038747	-2.521305
H	0.829139	5.054589	-3.572777
H	0.756349	6.010719	-2.061599
H	1.028418	4.240197	-1.988037
C	1.676307	5.299152	1.049931
H	2.374309	4.646508	0.502462

H	1.274759	6.175932	0.516736
C	1.346130	5.053686	2.339410
H	-1.072238	3.317119	1.801659
C	1.898969	3.874219	3.097125
H	1.079338	3.189158	3.405887
H	2.620545	3.290093	2.495834
H	2.403006	4.206026	4.030857
C	0.393527	5.934683	3.104700
H	0.871177	6.330154	4.027503
H	0.037720	6.792517	2.501385
H	-0.492834	5.350318	3.436009

TIBA-SiOH-INT2-AlH **(b4”)** E=-7065.070857 A.U.

C	1.678917	5.023178	-0.661656
C	1.005333	5.589563	0.610023
C	-0.491407	5.853265	0.384371
Al	1.110679	3.258078	-1.355357
O	-0.193094	2.440266	-0.466304
Si	-1.565224	1.601373	-0.623747
O	-1.495061	0.482457	-1.856543
Si	-1.859986	-1.128843	-1.935862
O	-2.282243	-1.954378	-0.576826
Si	-2.527356	-2.865085	0.776491
O	-2.096152	-1.846657	2.017190
Si	-1.884941	-0.199884	2.075019
O	-1.950350	0.632808	0.665344
O	-3.184400	0.415281	2.914284
Si	-4.702174	0.592998	2.178836
O	-5.181371	-0.842597	1.485282
Si	-5.388075	-2.173172	0.510270
O	-4.146072	-3.245502	0.848321
O	-4.734159	1.737313	0.969974
Si	-4.459638	2.082725	-0.641177
O	-2.883787	2.598442	-0.843064
O	-4.688917	0.749349	-1.613897
Si	-4.753065	-0.766277	-2.291336
O	-5.340384	-1.786244	-1.110126
O	-3.233644	-1.244121	-2.869409
O	-0.304520	-0.033574	2.593772
Si	1.000442	-0.443114	1.652364
O	1.972130	0.890963	1.528401
Si	3.087974	1.853786	0.803864
O	2.691613	1.953576	-0.881040
Si	3.032636	0.851589	-2.183841
O	4.485004	0.138042	-1.858789
Si	5.470515	-1.147137	-1.463285
O	6.078910	-0.846544	0.057867
Si	5.488777	-0.130378	1.440070
O	4.599205	1.216768	0.991254
O	1.849475	-1.646951	2.405961
Si	3.341653	-2.374474	2.278153
O	3.414594	-3.258097	0.873346
Si	3.340126	-3.414827	-0.778562
O	1.884994	-2.833916	-1.340154
Si	0.960777	-1.466947	-1.436068
O	-0.385490	-1.816943	-2.346577
O	0.585767	-0.958402	0.115526
O	1.827321	-0.270909	-2.193412
O	4.520184	-1.196871	2.260707
O	4.585809	-2.550710	-1.470475
C	1.711513	6.862245	1.104859
H	3.544845	-3.266458	3.440286
H	-5.637226	0.993682	3.255104
H	-5.382628	3.166954	-1.051056
H	-5.667644	-0.790338	-3.455645
H	3.028110	3.234675	1.322850
H	3.123575	1.607387	-3.444528
H	3.464241	-4.839959	-1.153331
H	-6.683825	-2.825958	0.809668
H	-1.730016	-4.113909	0.814656
H	6.616419	0.292031	2.299909
H	6.583702	-1.261647	-2.431422
H	2.786509	5.006214	-0.530446
H	1.521467	5.745302	-1.499450
H	1.086793	4.828567	1.421834
H	1.246228	7.258270	2.032291
H	2.784964	6.676513	1.317920
H	1.660547	7.663384	0.336024
H	-0.977929	6.241879	1.302940
H	-0.639434	6.611384	-0.415337
H	-1.032105	4.933219	0.082837
H	1.121350	2.918833	-2.922586

TIBA-SiOH-TS2-AlH **(b4”-b5”)** E=-7065.054152 A.U.

C	-1.720646	5.138705	-0.630623
C	-0.574970	6.096980	-0.234607
C	-0.301466	6.050448	1.275979
Al	-1.437628	3.246995	-0.210998
O	0.121727	2.523977	-0.490763
Si	1.479909	1.680932	-0.212800
O	1.649928	0.293188	-1.098377
Si	1.819177	-1.090614	-1.981464
O	3.143172	-0.783777	-2.943311
Si	4.678187	-0.357439	-2.381175
O	5.204292	-1.385558	-1.184137
Si	5.442417	-2.192841	0.251299
O	5.383553	-1.180219	1.576967
Si	4.752772	0.205547	2.258943
O	3.242237	-0.019853	3.002024
Si	1.904363	-0.310114	2.050340
O	2.468533	-1.488041	1.053463
Si	2.616698	-2.942743	0.281728
O	2.152036	-2.554834	-1.260354
O	-2.745370	2.021537	-0.462308
Si	-2.861689	1.642106	1.727249

O	-4.360760	1.021332	1.326653
Si	-5.384367	-0.275637	1.530198
O	-6.008176	-0.703938	0.048512
Si	-5.505504	-0.804074	-1.535728
O	-4.590327	0.541773	-1.891106
Si	-3.055619	1.160169	-1.868426
O	-1.956041	-0.067746	-1.954848
Si	-1.025431	-1.362430	-1.560705
O	0.286274	-1.360119	-2.588949
O	-1.668589	0.498394	1.696504
Si	-0.909399	-0.962602	1.643046
O	-1.888183	-2.149151	2.245295
Si	-3.410624	-2.764965	1.949253
O	-3.478506	-3.346575	0.396350
Si	-3.435693	-3.250219	-1.262562
O	-4.602888	-2.179830	-1.771527
O	-0.545292	-1.295866	0.045983
O	0.449940	-0.909436	2.615096
O	-1.928020	-2.737138	-1.765821
C	-0.856517	7.534716	-0.697589
O	2.813240	2.639639	-0.473781
Si	4.402608	2.136724	-0.401239
O	4.707583	1.170970	-1.724093
O	1.507180	1.136683	1.358322
O	4.637508	1.324784	1.032774
O	4.230742	-3.346091	0.370861
O	-4.534571	-1.557545	2.167862
H	-3.691203	-4.584482	-1.849119
H	5.584458	-0.411062	-3.550657
H	5.289442	3.322201	-0.458621
H	5.668470	0.677209	3.323157
H	-2.887965	2.071894	-3.031565
H	-3.099005	2.044397	3.156640
H	-3.668206	-3.862374	2.907767
H	6.758321	-2.873294	0.236203
H	1.801171	-4.046372	0.841617
H	-6.692073	-0.838357	-2.420209
H	-6.504436	0.065385	2.438504
H	-1.902844	5.195752	-1.729532
H	-2.676100	5.471458	-0.161825
H	0.351149	5.752279	-0.749107
H	-0.014953	8.215801	-0.452275
H	-1.020822	7.582435	-1.793598
H	-1.768473	7.937265	-0.205947
H	0.522299	6.734413	1.565851
H	-1.202333	6.347479	1.855334
H	-0.008229	5.031054	1.610818
H	-2.066892	3.013185	1.480832

References

(1) Sommer, W.; Gottwald, J.; Demco, D. E.; Spiess, H. W. *Journal of Magnetic Resonance, Series A* **1995**, *113*, 131.

(2) Rataboul, F.; Baudouin, A.; Thieuleux, C.; Veyre, L.; Copéret, C.; Thivolle-Cazat, J.; Basset, J.-M.; Lesage, A.; Emsley, L. *J. Am. Chem. Soc.* **2004**, *126*, 12541.

(3) Bax, A.; Griffey, R. H.; Hawkins, B. L. *Journal of Magnetic Resonance (1969)* **1983**, *55*, 301.

(4) Frisch, M. J.; Trucks, G. W.; Schlegel, H. B.; Scuseria, G. E.; Robb, M. A.; Cheeseman, J. R.; Scalmani, G.; Barone, V.; Mennucci, B.; Petersson, G. A.; Nakatsuji, H.; Caricato, M.; Li, X.; Hratchian, H. P.; Izmaylov, A. F.; Bloino, J.; Zheng, G.; Sonnenberg, J. L.; Hada, M.; Ehara, M.; Toyota, K.; Fukuda, R.; Hasegawa, J.; Ishida, M.; Nakajima, T.; Honda, Y.; Kitao, O.; Nakai, H.; Vreven, T.; Montgomery Jr., J. A.; Peralta, J. E.; Ogliaro, F.; Bearpark, M. J.; Heyd, J.; Brothers, E. N.; Kudin, K. N.; Staroverov, V. N.; Kobayashi, R.; Normand, J.; Raghavachari, K.; Rendell, A. P.; Burant, J. C.; Iyengar, S. S.; Tomasi, J.; Cossi, M.; Rega, N.; Millam, N. J.; Klene, M.; Knox, J. E.; Cross, J. B.; Bakken, V.; Adamo, C.; Jaramillo, J.; Gomperts, R.; Stratmann, R. E.; Yazyev, O.; Austin, A. J.; Cammi, R.; Pomelli, C.; Ochterski, J. W.; Martin, R. L.; Morokuma, K.; Zakrzewski, V. G.; Voth, G. A.; Salvador, P.; Dannenberg, J. J.; Dapprich, S.; Daniels, A. D.; Farkas, Ö.; Foresman, J. B.; Ortiz, J. V.; Cioslowski, J.; Fox, D. J.; Gaussian, Inc.: Wallingford, CT, USA, 2009.

(5) Perdew, J. P.; Burke, K.; Ernzerhof, M. *Physical Review Letters* **1996**, *77*, 3865.

(6) Perdew, J. P.; Burke, K.; Ernzerhof, M. *Physical Review Letters* **1997**, *78*, 1396.

(7) Schlegel, H. B. *Journal of Computational Chemistry* **1982**, *3*, 214.

(8) Adamo, C.; Barone, V. *The Journal of Chemical Physics* **1999**, *110*, 6158.

(9) Schäfer, A.; Horn, H.; Ahlrichs, R. *The Journal of Chemical Physics* **1992**, *97*, 2571.

(10) Schäfer, A.; Huber, C.; Ahlrichs, R. *The Journal of Chemical Physics* **1994**, *100*, 5829.

(11) Rozanska, X.; Delbecq, F.; Sautet, P. *Physical Chemistry Chemical Physics* **2010**, *12*, 14930.

(12) Solans-Monfort, X.; Chow, C.; Gouré, E.; Kaya, Y.; Basset, J.-M.; Taoufik, M.; Quadrelli, E. A.; Eisenstein, O. *Inorganic Chemistry* **2012**, *51*, 7237.

(13) Samantaray, M. K.; Callens, E.; Abou-Hamad, E.; Rossini, A. J.; Widdifield, C. M.; Dey, R.; Emsley, L.; Basset, J.-M. *J. Am. Chem. Soc.* **2014**, *136*, 1054.

(14) Riache, N.; Dery, A.; Callens, E.; Poater, A.; Samantaray, M.; Dey, R.; Hong, J.; Li, K.; Cavallo, L.; Basset, J.-M. *Organometallics* **2015**, *34*, 690.

(15) Pasha, F. A.; Cavallo, L.; Basset, J. M. *ACS Catal.* **2014**, *4*, 1868.

(16) D'Elia, V.; Ghani, A. A.; Monassier, A.; Sofack-Kreutzer, J.; Pelletier, J. D. A.; Drees, M.; Vummaleti, S. V. C.; Poater, A.; Cavallo, L.; Cokoja, M.; Basset, J.-M.; Kühn, F. E. *Chem. - Eur. J.* **2014**, *20*, 11870.

(17) Chen, Y.; Credendino, R.; Callens, E.; Atiqullah, M.; Al-Harthi, M. A.; Cavallo, L.; Basset, J.-M. *ACS Catal.* **2013**, *3*, 1360.
